# Cluster-based network proximities for arbitrary nodal subsets

**DOI:** 10.1038/s41598-018-32172-0

**Published:** 2018-09-25

**Authors:** Kenneth S. Berenhaut, Peter S. Barr, Alyssa M. Kogel, Ryan L. Melvin

**Affiliations:** 1 Department of Mathematics and Statistics, Wake Forest University, Winston-Salem, NC 27109 USA; 2 Department of Computer Science, Wake Forest University, Winston-Salem, NC 27109 USA; 3 Present Address: Department of Statistics, West Virginia University, Morgantown, WV 26506 USA; 4 Department of Physics, Wake Forest University, Winston-Salem, NC 27109 USA

## Abstract

The concept of a *cluster* or *community* in a network context has been of considerable interest in a variety of settings in recent years. In this paper, employing random walks and geodesic distance, we introduce a unified measure of cluster-based proximity between nodes, relative to a given subset of interest. The inherent simplicity and informativeness of the approach could make it of value to researchers in a variety of scientific fields. Applicability is demonstrated via application to clustering for a number of existent data sets (including multipartite networks). We view community detection (i.e. when the full set of network nodes is considered) as simply the limiting instance of clustering (for arbitrary subsets). This perspective should add to the dialogue on what constitutes a cluster or community within a network. In regards to health-relevant attributes in social networks, identification of clusters of individuals with similar attributes can support targeting of collective interventions. The method performs well in comparisons with other approaches, based on comparative measures such as NMI and ARI.

## Introduction

**Figure 1:**
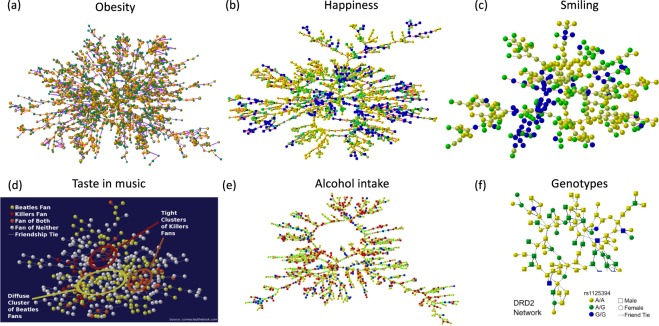
Examples of social networks with noted *clustering* of nodes of interest. The figures have been reproduced by permission of the authors of the respective manuscripts; for further details see the references as indicated. (**a**) A network of individuals in 2000 from the Framingham Heart Study (FHS) Social Network^9^. Connections arise from friendship, marital and familial ties. Yellow nodes indicate individuals with body mass index greater than or equal to 30, and nodes are colored green otherwise. The size of each node is proportional to the individual’s body-mass index. The authors of^9^ note that clusters of obese and non-obese individuals are visible in the network. (**b**) A network of individuals in 1996 from the FHS Social Network^3^. Colors indicate mean happiness of egos and all directly connected alters, on a spectrum from blue (unhappy) to yellow (happy). Happiness is measured via the Center for Epidemiological Studies depression scale. The authors of^3^ note that “clusters of happy and unhappy people are visible in the network”. (**c**) A social network of individuals in 2007 ascertained using Facebook^21^. Ties indicate the connected individuals were tagged in a photo together. Yellow nodes reflect individuals who are smiling in profile photographs and surrounded by others who are smiling. Similarly blue nodes reflect individuals who are frowning, surrounded by others who are frowning, and green indicate a mix of smiling and non-smiling friends. The graph suggests clustering of both blue and yellow nodes. In addition those who do not smile appear to be more scattered towards the peripherally in the network. (**d**) A social network of individuals in 2007 whose social ties were ascertained via Facebook^6^. The interior color of the nodes indicates the individual’s taste in music. The graph suggests clustering (both diffuse and closely-knit) based on musical tastes within the network. (**e**) A network of individuals in 2000 from the FHS Social Network^2^. Node color denotes the alcohol intake of the subject, with red indicating an abstainer and blue indicating heavy intake (yellow nodes indicate moderate intake). As noted by the authors of^2^, “the graph suggests clustering in abstention and heavy alcohol consumption behavior”. (**f**) A network of individuals from the National Longitudinal Study of Adolescent Health (Add Health) Social Network^76^, started in 1994^77^. Node color indicates genotypes for DRD2 (which has been associated with alcoholism). The graph suggests clustering of genotypes.

There has been heightened interest recently regarding clustering of individuals in social networks based on characteristics such as tobacco use^1^, alcohol consumption^2^, level of happiness^3^, emotion^4^, divorce^5^, cultural preferences^6,7^, gun violence^8^, and general health behaviors and attitudes^9–15^ (see Fig. 1). However, there is little notion of what definitively constitutes a tightly or diffusely knit cluster in such instances. The requirement that individuals comprising a cluster be linked via path-wise attachment (through nodes of similar characteristic) may not be appropriate, particularly in cases where there may be missing data regarding links or nodal attributes. Here we provide a notion of proximity of nodes restricted to a subset for a network, which is then well-suited for analysis via extant clustering procedures. The method can be applied with informativeness through all levels of subset size, from only a few nodes in a large network through consideration of the limiting case of all nodes (commonly referred to as *community detection* or *graph partitioning*; see for instance Porter *et al*.^16^, Newman^17^, Schaeffer^18^ and Fortunato^19,20^). The work here has applications in scientific fields where networks with nodal attributes arise including biology, ecology, neuroscience, physics, computer science, sociology, psychology, chemistry, and economics. One side benefit of the approach is that, applied in community detection, it is parsimonious and simple (see Eq. 1). To the best of our knowledge this is the first work specifically providing a measure of proximity between nodes that adequately reflects cluster membership for restriction to arbitrary nodal subsets on arbitrary networks (including non-spatial networks; see Related Work). This should add to the dialogue on what constitutes a community within a network. As mentioned in^21^, in regards to health-relevant attributes in social networks, identification of cliques or clusters of individuals with similar attributes can support targeting of collective interventions.

The remainder of the paper proceeds as follows. We first introduce the concept of community-relative distance (see Community-relative Distance), and then turn to discussion of applications in the context of related work (see Related Work and Applications and Discussion). The paper ends with some technical computational considerations (see Materials and Methods).

## Community-Relative Distance

**Figure 2:**
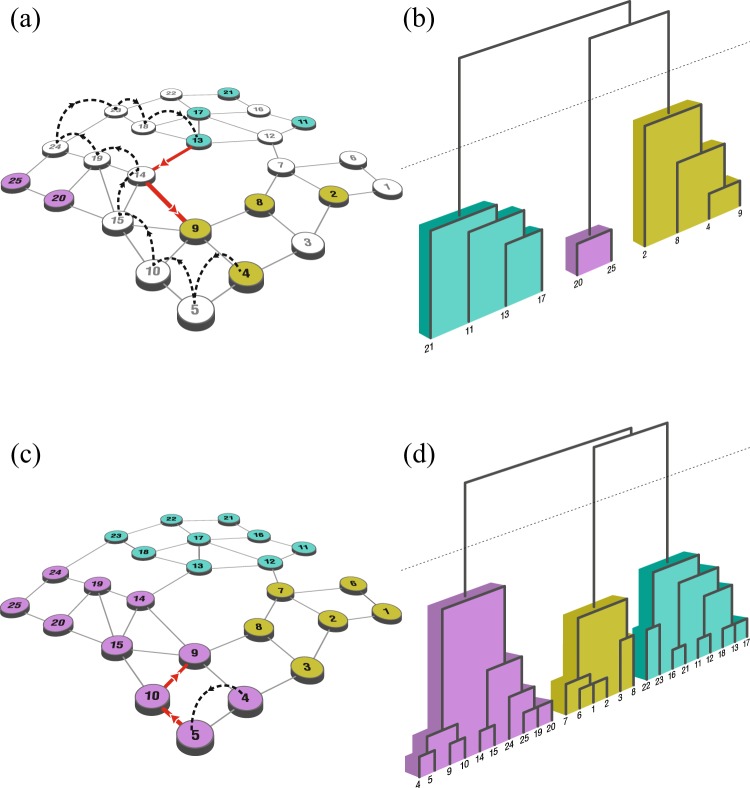
(**a**,**b**) A subset of ten selected nodes within a larger network of 25 nodes. A sample path for a random walk departing from node 4, and eventually entering the set of selected nodes at node 13 is indicated with dashed lines. The solid red line indicates a shortest path to the “target” node, node 9. A resulting dendrogram (via average-linkage clustering) is given in (**b**). A separation into three distinct clusters can be seen in the dendrogram. (**c**,**d**) The 25 node network with *S* comprised of all 25 nodes. A one-step sample path for a random walk departing from node 4, entering the set *S* at node 5 is indicated with dashed lines. The solid red line indicates a shortest path of length two to node 9. A resulting dendrogram is given in (**d**). A separation into three clusters (communities) can again be seen in the dendrogram.

Consider a network represented as a graph, *G* = (*V*, *E*), with a set of vertices or nodes, *V*, and a set of edges, *E* (see Fig. 2 for an example of a 25-node, 40 edge graph). We assume the graph is connected and undirected, and the edges are unweighted (although it is not difficult to extend the work to weighted edges). Suppose some subset of nodes, *S*, is selected. These nodes could represent, for instance, infected individuals in a social network (or individuals with specific attributes, such as obesity or other health behaviors), suspected terrorists in a communication network, crimes on a spatial city street network, genes and conditions in gene expression networks, disease-related genes, proteins, or metabolites in an interaction network, etc., or simply nodes of high degree in a larger network.

The underlying idea, here (see the example in Fig. 2), is that if two nodes, *i* and *j*, are part of a closely-knit community, then an individual resting at node *i* and perturbed off the node should encounter first (via randomly walking) a subset node “close” to node *j*. Specifically, for any two nodes, *i* and *j* in *S*, consider a random walk on *G* departing from node *i*. We define the *distance from i to j* (relative to the members of *S*), as the expected shortest-path (or geodesic) distance to *j* of the first node in *S* that the random walk encounters. We denote the resulting |*S*| × |*S*| matrix of distances as ***D***.

Now, define the *distance between i and j* (again, relative to the members of *S*) to be the smaller of the two associated distances (*i* to *j* and *j* to *i*), with the intuition that connections can be asymmetrically initiated (see Remark 1, below). Note that the reflexive distance between node *i* and itself is taken to be zero. In what follows, for convenience, we will refer to the resulting symmetric |*S*| × |*S*| matrix of distances as D^*^ (given for the example in Fig. 2a in Fig. S1), and the individual entries as *community-relative distances*. The reader is referred to Lovász^22^ and Aldous and Fill^23^ for discussion of random walks on graphs, and Pons and Latapy^24^, Zhou and Lipowsky^25^, and Zhou^26^ for some discussion in the context of community detection (see also Related Work, below). For a survey on distance measures on graphs, see^27^, and the references therein; for discussion of kernel-based measures, see for instance^28^. In general, one could replace the shortest-path distances used here with another context-dependent measure (including ***D***, in an iterative fashion).

### **Remark 1**.

Note that the distances from *i* to *j* and *j* to *i* may be quite different. For the network in Fig. 2(a), consider a random walk departing from node 4. The expected distance to node 9 of the first node in *S* that the walk encounters is 0.83 (reflecting likely encounters with nodes proximate to 9, such as 8 and 9, itself). On the other hand, for a random walk departing from node 9, the corresponding expected distance to node 4 is 1.52 (reflecting potential encounters with nodes distant to 4 such as 13 and 20).

### **Example 1**.

**Figure 3:**
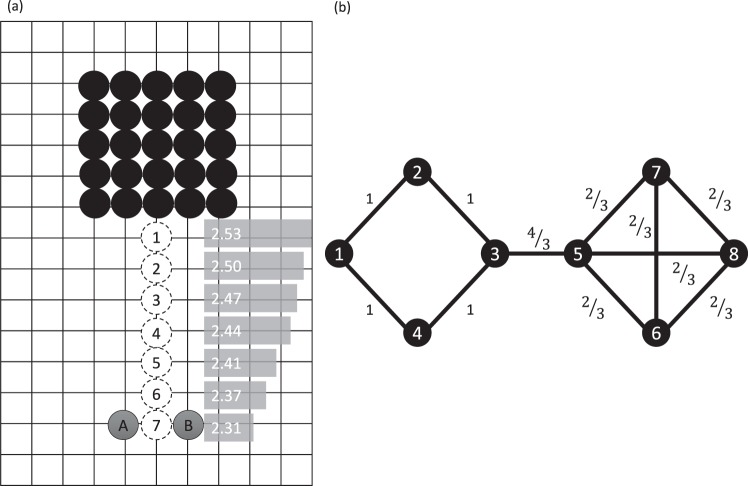
(**a**) A 15 × 11 grid with set *S* consisting of the 5 × 5 sub-grid at the top (in black) as well as the two nodes A and B. The community-relative distance between A and B is 2.55. The updated distance when a single node is added at one of the locations denoted 1 through 7, is given to the right of the respective location (see Example 1). Note that as the extra node approaches A and B, the two nodes become more proximate, in a sense shrinking the space between A and B, as they become part of a stronger community. (**b**) A simple 8-node graph. Community-relative distances for nodes at shortest-path distance one are indicated adjacent to the corresponding edge. Note that the tie between node 3 and node 5 is one of greater community-relative distance, suggesting separation, while the ties in the clique consisting of nodes 5–8 correspond to smaller community-relative distance (see Example 2).

Figure 3a gives a dynamic perspective, which illustrates the connection between community-relative distances as suggested above and cluster membership. Consider the 165-node, 15 × 11 grid graph, *G*, with nodal subset *S* consisting of the 25 nodes in the 5 × 5 sub-grid in black, as well as the two nodes A and B shaded grey. For a random walk departing from Node A, the expected distance to B of the first node in *S* encountered is 2.55, as initial entry into *S* is likely to occur at a black node distant to B. Now, consider *S* augmented by the node at the position labeled 1; the expected distance now shrinks slightly to 2.53. The expected distances for the 28-node subsets obtained by augmenting with nodes 2 through 7 (in turn, in place of 1), are indicated adjacent to the corresponding node. Note that as the additional node is moved from positions 1 through 7, nodes A and B are in a sense drawn closer together, as the distance between them shrinks from 2.55 (with no added node) to 2.31 (for an added node at position 7). In a more general sense, two nodes at fixed position in a network will be drawn closer in community-relative distance when there are other proximate network nodes in the subset of interest. The scenario reflects a *cooperation* pattern between nodes in a community, and the leveraging of distance-based information from proximate nodes, particularly through weak ties^29^.$\square$

### **Example 2**.

Figure 3b provides a simple example of an 8-node graph, with community-relative distances for pairs at shortest-path distance one indicated adjacent to the corresponding edge. Note that the tie between node 3 and node 5 is one of greater community-relative distance, suggesting separation, while the ties in the clique consisting of nodes 5–8 correspond to smaller community-relative distance.$\square$

Community-relative distances could have potential use in missing link or attribute prediction, wherein small distances between unconnected nodes could suggest potential edges, and a large cluster of mixed attribute nodes could suggest missing attributes.

Employing D^*^, it is possible to cluster the elements of *S* via standard extant procedures. Unless specified otherwise, the results in what follows here arise from employment of average-linkage hierarchical clustering on D^*^, i.e. sequentially combining two clusters with the lowest average distance between pairs (see for instance Lazega *et al*.^30^ and Newman^31^ for discussion of hierarchical clustering). As mentioned in Related work below (see also Figs 3b, S2 and S3 and Applications and Discussion), community-relative distances reflect separation between clusters, and provide robust results under different clustering procedures (see Fig. S3). It can be worthwhile to look at the resulting dendrograms for overall clustering patterns and, if desired, natural locations to “cut” *S* into clusters (see Fig. 2b,d). There are several methods available for searching for appropriate dendrogram cut-points (see for instance^32^). For comparison purposes, below, to estimate reasonable stopping conditions, we employ an average silhouette width criterion (ASW; see^33^) as well as the variance ratio criterion (VR) of Caliński and Harabasz^34^ (see Related Work as well as Figs S4 and S5); similar results are obtained using other common extant methods. Also available are non-hierarchical methods such as partitioning around medoids (see^35^).

Importantly, note that in the case where *S* = *V*, i.e. all (say *n*) nodes are selected, the community-relative distance from node *i* to node *j*, described above, reduces to simply the average of the shortest-path distances from the direct neighbors of *i* to *j*. In fact, computation reduces simply to

1 $${\boldsymbol{D}}={\boldsymbol{P}}{{\boldsymbol{D}}}_{s}-{\boldsymbol{I}},$$

where ***P*** is the transition matrix for a random walk on the graph *G*, ***D***_*s*_ is the matrix of shortest path distances and ***I*** is the identity matrix (of size *n*). For further details and discussion of computational complexity in the general case, see Materials and Methods, below.

## Related Work

**Figure 4:**
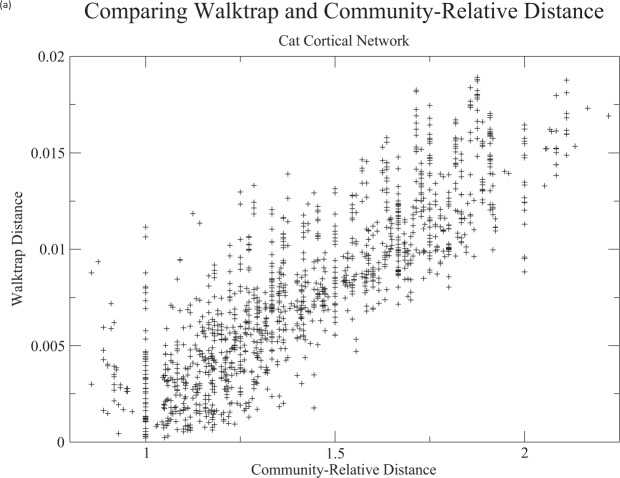
A plot of Walktrap (t = 4) distances against community-relative distances for the cat cortical network.

Closest to the work presented here, specifically in the limiting case of community detection, is the popular *Walktrap* method of Pons and Latapy^24^. Therein, random walks are also employed to obtain distances which can then be used in agglomerative hierarchical procedures. In particular, therein, the distance, *r*_*i*,*j*_ between nodes *i* and *j* is defined for fixed *t* ∈ {1, 2, …} via

2 $${r}_{i,j}(t)=\parallel {{\boldsymbol{\Delta }}}^{-1/2}{{\boldsymbol{P}}}_{i,\cdot }^{t}-{{\boldsymbol{\Delta }}}^{-1/2}{{\boldsymbol{P}}}_{j,\cdot }^{t}\parallel ,$$

where **Δ** is a diagonal matrix with diagonal entries Δ_*i*,*i*_ = *d*(*i*), *d*(*i*) is the degree of *v*_*i*_, ${{\boldsymbol{P}}}_{l,\cdot }^{t}$ is the column probability vector ${({P}_{l,k}^{t})}_{1\le k\le n}$, ***P*** = [*P*_*i*,*j*_] is the transition matrix for a random walk on the graph *G*, and |·| indicates the Euclidean norm on ${{\mathbb{R}}}^{n}$. A plot of these distances against community-relative distances for a cat cortical network (see^36^ and Applications and Discussion, below) is given in Fig. 4. Note that the ordering of distances is quite different in the two cases. In terms of community detection, community-relative distance does have advantages: (i) there is no need to choose an appropriate parameter *t*. The Walktrap method can be sensitive to values of *t*, as well as the choice of agglomerative method (compare Figs S2 and S3). (ii) Community-relative distances are particularly simple and parsimonious (See Eq. 1), while computational times are similar for the two methods, (iii) units of resulting distances are easily interpretable in terms of shortest path distance and (iv) most importantly, there is no immediate counterpart to clustering restricted to subsets in the case of the Walktrap algorithm.

**Figure 5:**
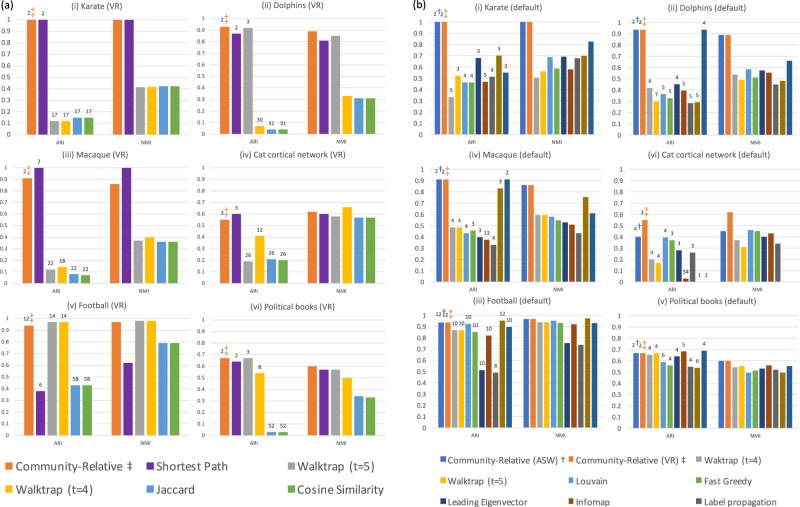
(**a**) ARI and NMI values for agglomerative clustering (employing average-linkage and a VR stopping condition) for some common networks possessing reasonable ground truths, via a range of common distance measures. The networks are discussed further in Applications and Discussion, below. For discussion of Jaccard and cosine similarity measures, see for instance^39^ and the references therein. (**b**) ARI and NMI values for the six network data sets, employing nine methods built into the *igraph* package in R (see^40^), alongside those for community-relative distance using both ASW and VR stopping conditions.

Figure 5a contains adjusted Rand index (ARI; see^37^), and normalized mutual information (NMI; see^38^) values for agglomerative clustering (employing average-linkage and a VR stopping condition) for some common networks possessing reasonable ground truths, via a range of common distance measures; for discussion of Jaccard and cosine similarity measures, see for instance^39^ and the references therein. Note that community-relative distance performs comparably or considerably better for the six common networks considered. For similar results employing ASW, see Fig. S4. The networks are discussed further in Applications and Discussion, below.

Figure 5b contains ARI and NMI values for the six network data sets, employing nine methods built into the *igraph* package in R (see^40^), alongside those for community-relative distance using both ASW and VR stopping conditions. Again community-relative distance performs comparably or considerably better for the networks considered. Plots and dendrograms under community-relative distance are provided in Applications and Discussion, below; for plots of associated ASW and VR values, see Fig. S5. For general discussion regarding comparing clusterings see for instance^41^. For other work related to community detection and random walks see^25^ and^26^ and the references therein.

In terms of restriction to nodal subsets, there has been considerable work recently in the special case of types within bipartite networks (see^42–51^). For discussion of community-relative distance in this context see Applications and Discussion, below. It is important to note that, contrasted with methods specific to bipartite networks, the perspective proposed here imposes no assumptions on the edge structure of the network considered, nor the sets under consideration for clustering.

For some recent work on attributes in the context of clustering, see^52^. Although different in scope, it is worth noting connected work on clustering in spatial networks (see for instance^53^). Community-relative distance is applicable for arbitrary (potentially non-spatial) networks, and may be of some potential future use in existing algorithms for spatial networks, in place of often considered geodesic distance. In addition, there has been important recent work employing stochastic complementation^54^ in the context of restriction to subsets of network nodes (see^55^ and [^28^, Section 10.4.5]).

## Applications and Discussion

In this section, we consider community-relative distance applied to several data sets, first in the context of proper nodal subsets, *S*, of interest, and finally in the context of community detection.

### Nodal subsets

**Figure 6:**
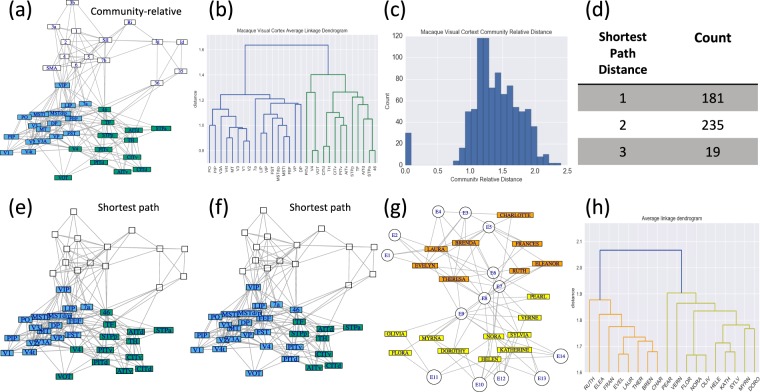
(**a**,**b**) An application of community-relative distance to the 30-node visual cortex subset within the 45-node cortical pathways network of the macaque monkey (see^56^). Note that we employ the force-directed layout algorithm of Fruchterman and Reingold^78^, throughout for network plots. (**c**,**d**) A histogram of community-relative distances is given in (**c**). The 435 distinct pairs of subset nodes are comprised of 181 at (shortest-path) distance one, 235 at distance two and 19 at distance three (see (**d**)). (**e**,**f**) A cut into two clusters using average linkage hierarchical clustering and shortest-path distances for the 30-node visual cortex, for two permutations of the vertex order. Note the sensitivity to vertex order. (**g**,**h**) Two-clustering (via community relative distances and average-linkage clustering) for the nodes representing the 18 women in the bipartite network of Davis *et al*.^58^.

In Fig. 6a–f, we consider a macaque cortical network^56^. Employing community-relative distances and average-linkage clustering on the subset consisting of the cortical areas within the visual cortex, we obtain a fairly clear partition into two clusters as indicated in Fig. 6a,b. A histogram displaying community-relative distances is given in Fig. 6c (see also Fig. S13). For comparison, there are only three distinct shortest path distances: 1, 2, and 3; a tabulation of these is given in Fig. 6d (see also Fig. S14). If agglomerative clustering were to be implemented given the shortest path distances, final results could depend heavily on the choices when dealing with tied distances (see Fig. 6e,f). In Fig. S6 we provide a two-clustering for each of the two factions which arose for the karate club at a large state university studied by Zachary^57^. The corresponding D^*^ matrices are given in Figs S7 and S8, respectively.

**Figure 7:**
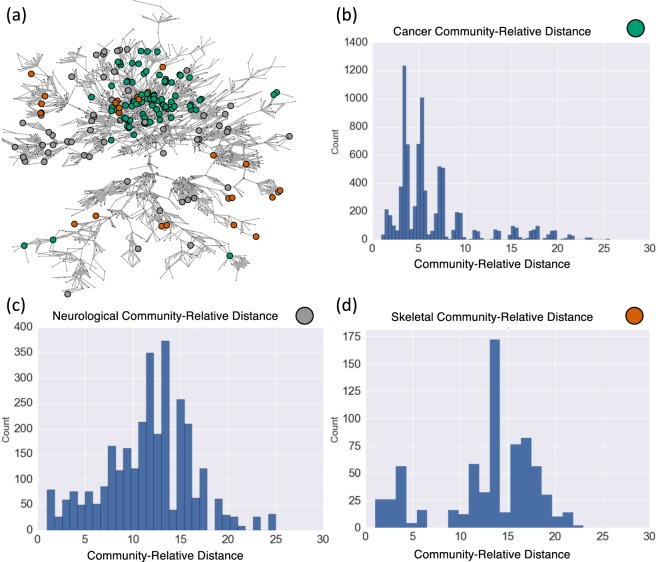
(**a**) The human disease network^62^. Here nodes corresponding to cancer, neurological and skeletal diseases are highlighted in green, grey and brown, respectively. (**b**–**d**) Histograms for the corresponding community-relative distances. For additional considerations for the human disease network, see Figs S11 and S12.

Since it is possible to consider any subset *S* contained in *V*, it is feasible to consider nodes of a particular type in a multipartite network. Davis *et al*.^58^ studied a group of 18 women and their observed participation in social events. Here we obtain the dendrogram in Fig. 6g,h. The suggested structural split into clusters (via ASW or VR) matches well with those in the meta-analyses of 21 studies as presented in^59^, Fig. 7; the match is exact with two of the methods considered therein. Similarly, consider the bipartite network of US Supreme court justice decisions for the 2000–2001 term^60^, depicted in Fig. S10. Here edges are drawn from each of the nine justices to any of 24 important cases for which they voted in the minority (two of the 26 cases from the original data had unanimous decisions). It is possible to consider the justices and cases, separately, by taking *S* as the set of justices, or the set of cases, respectively. Clusterings of the justices into 4 groups, and cases into 7 groups match exactly those as suggested in^60^, Fig. 1; see Fig. S10. For other discussion of analyzing community structures in two-mode (bipartite) networks, see for instance^61^ and the references, therein. As mentioned earlier, contrasted with extant methods specific to bipartite networks, the perspective proposed here imposes no assumptions on the edge structure of the network considered, nor the sets under consideration for clustering.

For an additional example, in Fig. 7 we consider three disease subsets from the human disease network^62^ consisting of disorders and the disease genes whose mutations are associated with the disorders. Histograms for community-relative distances for cancer, neurological and skeletal diseases are given in Fig. 7b–d, respectively. Note the distinct differences in the distributions of community-relative distances for the three disease node subsets. Cancer nodes are more closely positioned within the network; whereas, neurological and skeletal disease nodes are more diffusely positioned. Analyses informed by community-relative distance may aide in uncovering key cellular pathway components that lead to disease. A network plot and dendrograms for the three disorder classes are given in Figs S11 and S12.

### Community detection

**Figure 8:**
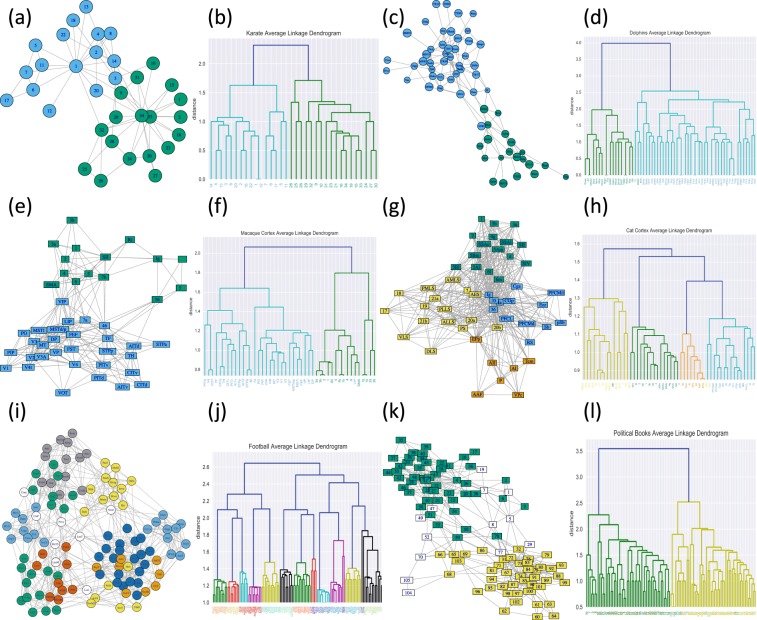
Graph plots and dendrograms (employing community-relative distance and average-linkage hierarchical clustering) for the six full networks considered in Fig. 5. (a,b) A two-clustering for the karate network of Zachary^57^. (**c**,**d**) A two-clustering clustering for the dolphin social network^63^. (**e**,**f**) A two-clustering for a 45-node cortical pathways network of the macaque monkey^56^. (**g**,**h**) A four-clustering for the cat cortical network^36^. (**i**,**j**) A twelve-clustering for the nodes representing the 2000–2001 NCAA football teams in the network of Girvan and Newman^65^. (**k**,**l**) A two-clustering for the political books network^66^. The nodes are colored to reflect apparent political affiliation (white for neutral, green for conservative and yellow for liberal), as suggested in^67^. Black is used in the dendrogram labels, in the neutral case, in place of white.

Figure 8a–l contains plots and dendrograms for the networks considered earlier from a numerical perspective in Related Work; for full numeric comparisons with other methods, see Fig. 5. For the case of the karate network (Fig. 8a,b), we obtain a two-clustering which captures the factions suggested in^57^ (ARI and NMI values of 1). As noted therein, Individual 9 was “a structural part”^57^ of the group assigned to in Fig. 8a; however, following fission of the original club, this individual did join the other group (due to some personal motivation). The corresponding full D^*^ matrix of community relative distances is given in Fig. S9.

In the case of the network produced by Lusseau from following a pod of dolphins in Doubtfull Sound, off the coast of New Zealand^63^ (Fig. 8c,d), we find results which nearly match the factions encountered in^64^ (2 nodes misclassified; an ARI value of 0.93; and NMI value of 0.89). In Fig. 8e,f, we consider the macaque brain network considered earlier. Here, we obtain a clear separation into two clusters which reflects membership in either the visual or sensorimotor cortices (1 node misclassified; ARI value of 0.91; and NMI value of 0.86). As a further example of community detection on a highly connected graph, we consider the cat cortical network discussed by Scannell *et al*. (see Fig. 8g,h)^36^. We find that the results obtained via community-relative distance is in strong agreement with the standard classification into four major thalamocortical systems^36^ (5 nodes misclassified; ARI value of 0.55; NMI value of 0.62).

Finally, for the case of community detection, we provide two additional examples. The first is a network with (American) collegiate football teams as nodes, and edges representing games played against one another^65^. Community-relative distance and average-linkage clustering (see Fig. 8i,j) quite clearly split these teams into the underlying conferences with accuracy (4 nodes misclassified – all independent teams; NMI value of 0.97; ARI value of 0.94). Some novel characteristics of the dendrogram may be noted, including the fact that many teams which later joined the Atlantic Coast Conference (ACC) are situated close to the ACC teams in the dendrogram. We also obtain similarly appropriate results with a network of politically themed books with edges connecting books commonly purchased together on amazon.com^66^. Here community-relative distance and average linkage clustering split the books quite well into groups of political affiliation, as identified by Newman^67^ (4 conservative or liberal nodes misclassified; ARI value of 0.67; and NMI value of 0.60).

#### **Remark 2**.

Note for the latter two examples in the section, results can be improved, if we restrict the set *S* to only nodes of interest. In the case of the football network, if we exclude consideration of independent teams (without conference membership), we obtain a perfect eleven-clustering into conferences (ARI and NMI values of 1). Similarly for the political blogs network, restricting *S* to the set of non-neutral books leads to only 3 books being misclassified.$\square$

## Materials and Methods

In this section we address computing of the (distance) entries in the matrix *D* = [*d*_*i*,*j*_]. Code in the R programming language is available upon request.

Suppose *G* = (*V*, *E*) and $S\subseteq V$ are fixed. Let ***A*** = [*A*_*i*,*j*_] be the adjacency matrix for *G*, i.e. *A*_*i*,*j*_ = 1 if (*v*_*i*_, *v*_*j*_) ∈ *E* and zero otherwise, ***I*** be the *n* × *n* identity matrix, and **Δ** be a diagonal matrix with diagonal entries Δ_*i*,*i*_ = *d*(*i*), where *d*(*i*) is the degree of *v*_*i*_.

For a general subset $S=\{{s}_{1},{s}_{2},\ldots ,{s}_{m}\}\subseteq V$, the matrix ***D*** can be obtained in the following manner. Define the matrix ***L*** = [*L*_*i*,*j*_] via

3 $${L}_{i,j}=\{\begin{array}{cc}1 & {\textstyle \text{if}\,i=j}\\ -1/d(i) & {\textstyle \text{if}\,{v}_{i}\notin S\,\text{and}\,({v}_{i},{v}_{j})\in E}\\ 0 & {\textstyle \text{otherwise}}\end{array}.$$

Note that ***L*** is similar to the random-walk normalized Laplacian matrix, L^*^ = ***I*** − **Δ**^−1^***A*** except that if *v*_*i*_ ∈ *S*, then the *i*-th row of L^*^ is replaced with *e*_*i*_ = (0, 0, …, 0, 1, 0 …, 0), i.e. the *i*-th row of the *n* × *n* identity matrix. Now, set

4 $$\hat{{\boldsymbol{D}}}={\boldsymbol{P}}{{\boldsymbol{L}}}^{-1}{\tilde{{\boldsymbol{D}}}}_{s},$$

where ${\tilde{{\boldsymbol{D}}}}_{s}$ is similar to the matrix of shortest path distances ***D***_*s*_, except that if *v*_*i*_ ∉ *S*, then the *i*-th row of ***D***_*s*_ is replaced with 0 = (0, 0, …, 0 …, 0), i.e. a null *n*-vector of zeros. The (*i*, *j*)-entry in $\hat{{\boldsymbol{D}}}$ is then the community-relative distance from node *i* to node *j* relative to the set *S*.

As suggested in Eq. (4), the process of computing community-relative distances requires (i) all pairs shortest-path distances between nodes in *S*, (ii) a solution to ${\boldsymbol{LX}}={\tilde{{\boldsymbol{D}}}}_{s}$, and (iii) computation of the product $\hat{{\boldsymbol{D}}}={\boldsymbol{PX}}$. Note that for (i), the full matrix of shortest-path distances (or an approximation, see for instance^68–70^) are often available, as these arise in standard preliminary network analyses (and elsewhere), even for relatively large networks. When this is not the case, some savings may be possible since only intra-set distances for *S* are required. For (ii), ***L*** can be viewed as the normalized random walk Laplacian for a directed variant of the graph *G*, wherein outgoing edges from nodes in *S* have been removed. Here, recently developed Laplacian solvers (see^71^) may be employed and computation can then be sub-quadratic in *n* (at least for sparse graphs). As |*S*| increases, the matrix ***L*** becomes increasingly sparse, and in the extreme case where *S* = *V*, we have that ***L*** is simply the identity matrix ***I***. Since, within each column of ***X***, one needs only solve for |*S*^*c*^| entries, computations can be reduced to

5 $$\tilde{O}((|{E}_{cc}{|}^{3/4}|{S}^{c}|+|{E}_{cc}||{S}^{c}{|}^{2/3})|S|),$$

where *E*_*cc*_ denotes the set of within-*S*^*c*^ edges and the $\tilde{O}$ notation suppresses polylogarithmic factors^?^. For the multiplication in (iii), note that to obtain the |*S*| × |*S*| matrix of within-*S* community-relative distances, we may consider the sparse multiplication of a |*S*| × *n* matrix $\tilde{{\boldsymbol{P}}}$ and an *n* × |*S*| matrix $\tilde{{\boldsymbol{X}}}$, where $\tilde{{\boldsymbol{P}}}$ consists of the |*S*| rows of ***P*** corresponding to the elements in *S*, and $\tilde{{\boldsymbol{X}}}$ consists of the |*S*| columns of ***X*** corresponding to the elements in *S*. Note that $\tilde{{\boldsymbol{P}}}$ contains |*E*_*S*._| non-zero entries, where *E*_*S*._ is the set of edges outgoing from *S*, and hence the number of operations is of order

6 $$O(|{E}_{S\mathrm{.}}||S|).$$

As mentioned earlier, community-relative distances provide expanded separation between clusters. We have employed average-linkage hierarchichal clustering, here, which has complexity *O*(|*S*|^2^) (see^72,73^), in an effort to show that even naive clustering procedures can work well. One may chose to employ ***D*** in other proximity-based methods, as appropriate in applications. For a discussion of exact and approximation methods, with savings in both time and space complexity, see^74,75^.

In the case *S* = *V*, as mentioned earlier, the matrix ***D*** has a simple form given via

7 $${\boldsymbol{D}}={\boldsymbol{P}}{{\boldsymbol{D}}}_{s}-{\boldsymbol{I}}.$$

## Electronic supplementary material

Supplementary Information

## Data Availability

The computations here were performed using the R programming language; a documented package which employs optimized routines in C++, is available upon request.
