# Supplementary material for: Cluster-based network proximities for arbitrary nodal subsets
Source: Sci Rep. 2018 Sep 25;8:14371. doi: 10.1038/s41598-018-32172-0 (PMC6156331; doi:10.1038/s41598-018-32172-0)
Supplement: Supplementary file 1 — Supplementary Information [file 41598_2018_32172_MOESM1_ESM.pdf]

Supporting Information for "Cluster-based network proximities for arbitrary nodal subsets"

Kenneth S. Berenhaut[a,1], Peter S. Barr[b], Alyssa M. Kogel[a,c], and Ryan L. Melvin[a,d]

[a]Department of Mathematics and Statistics, Wake Forest University, Winston-Salem, NC, 27109, USA
[b]Department of Computer Science, Wake Forest University, Winston-Salem, NC, 27109, USA
[c]Current Address: Department of Statistics, West Virginia University, Morgantown, WV, 26506
[d]Department of Physics, Wake Forest University, Winston-Salem, NC, 27109, USA

June 25, 2018

	2	4	8	9	11	17	18	20	21	25
**2**	0.00	2.01	1.60	2.29	3.07	2.91	3.55	4.18	3.89	4.94
**4**	2.01	0.00	1.38	0.83	4.11	3.33	3.55	2.72	4.85	3.31
**8**	1.60	1.38	0.00	1.36	3.15	2.96	3.26	2.94	3.80	3.25
**9**	2.29	0.83	1.36	0.00	3.50	3.25	2.90	1.99	3.85	2.32
**11**	3.07	4.11	3.15	3.50	0.00	1.39	2.29	4.42	1.89	4.66
**17**	2.91	3.33	2.96	3.25	1.39	0.00	1.01	3.49	1.29	3.50
**18**	3.55	3.55	3.26	2.90	2.29	1.01	0.00	3.11	2.07	3.26
**20**	4.18	2.72	2.94	1.99	4.42	3.49	3.11	0.00	4.39	0.95
**21**	3.89	4.85	3.80	3.85	1.89	1.29	2.07	4.39	0.00	4.12
**25**	4.94	3.31	3.25	2.32	4.66	3.50	3.26	0.95	4.12	0.00

Figure 1: The $|S| \times |S|$ matrix of community-relative distances, $\boldsymbol{D}^*$, for the example network in Figure 2a.

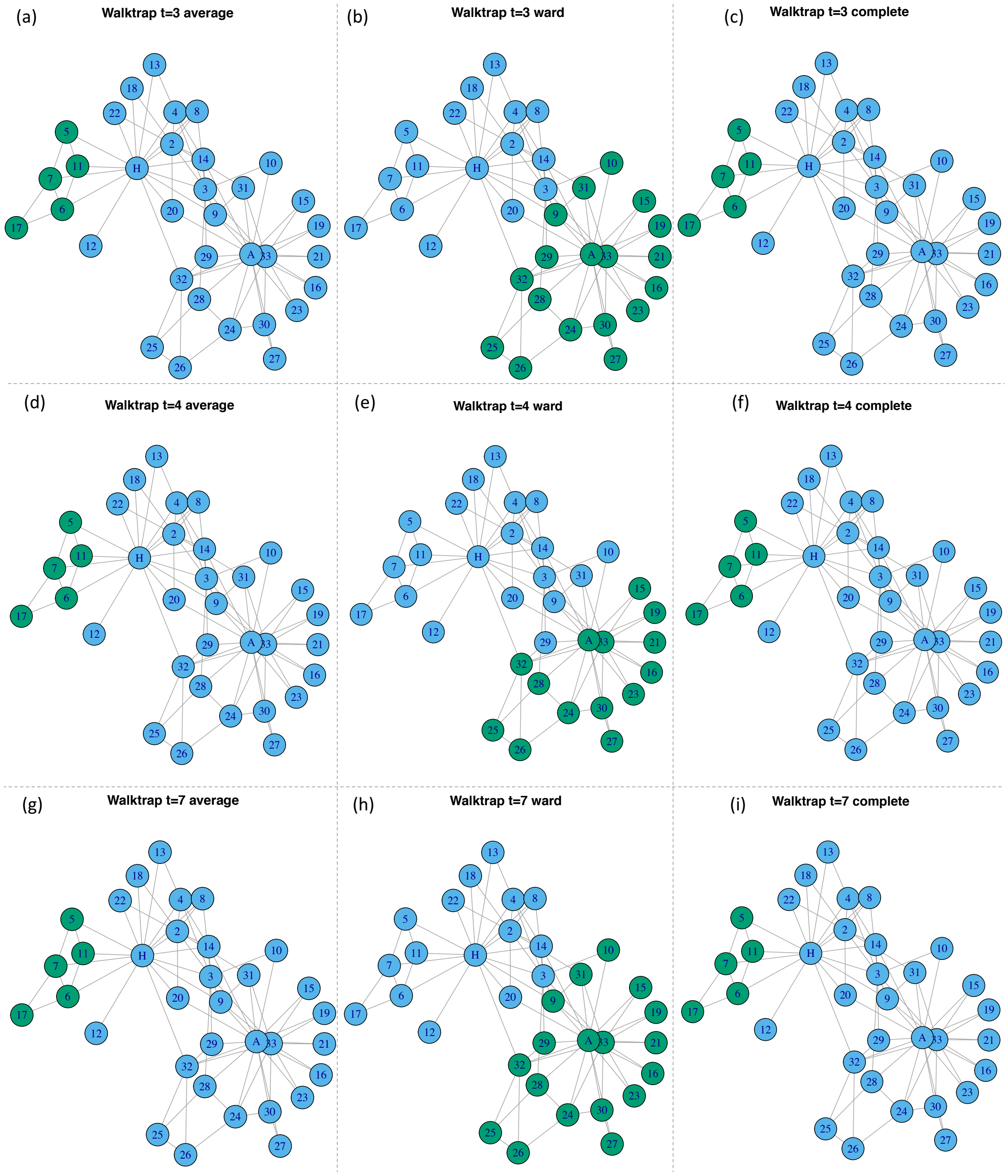

Figure 2: Two-clusterings of the karate club network [1] employing distances computed via the Walktrap method (for $t = 3$, $t = 4$ and $t = 7$) and average-linkage, Ward and complete-linkage agglomerative clustering. (a-c) $t = 3$, (d-f) $t = 4$ and (g-i) $t = 7$. Nodes are coded by cluster label.

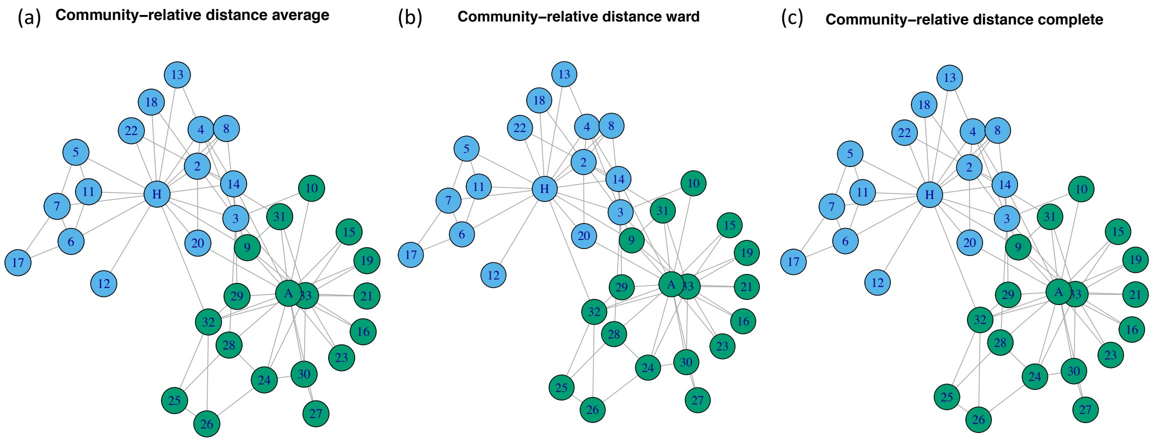

Figure 3: Two-clusterings of the karate club network [1] employing community-relative distances, and (a) average-linkage, (b) Ward and (c) complete-linkage agglomerative clustering. Nodes are coded by cluster label.

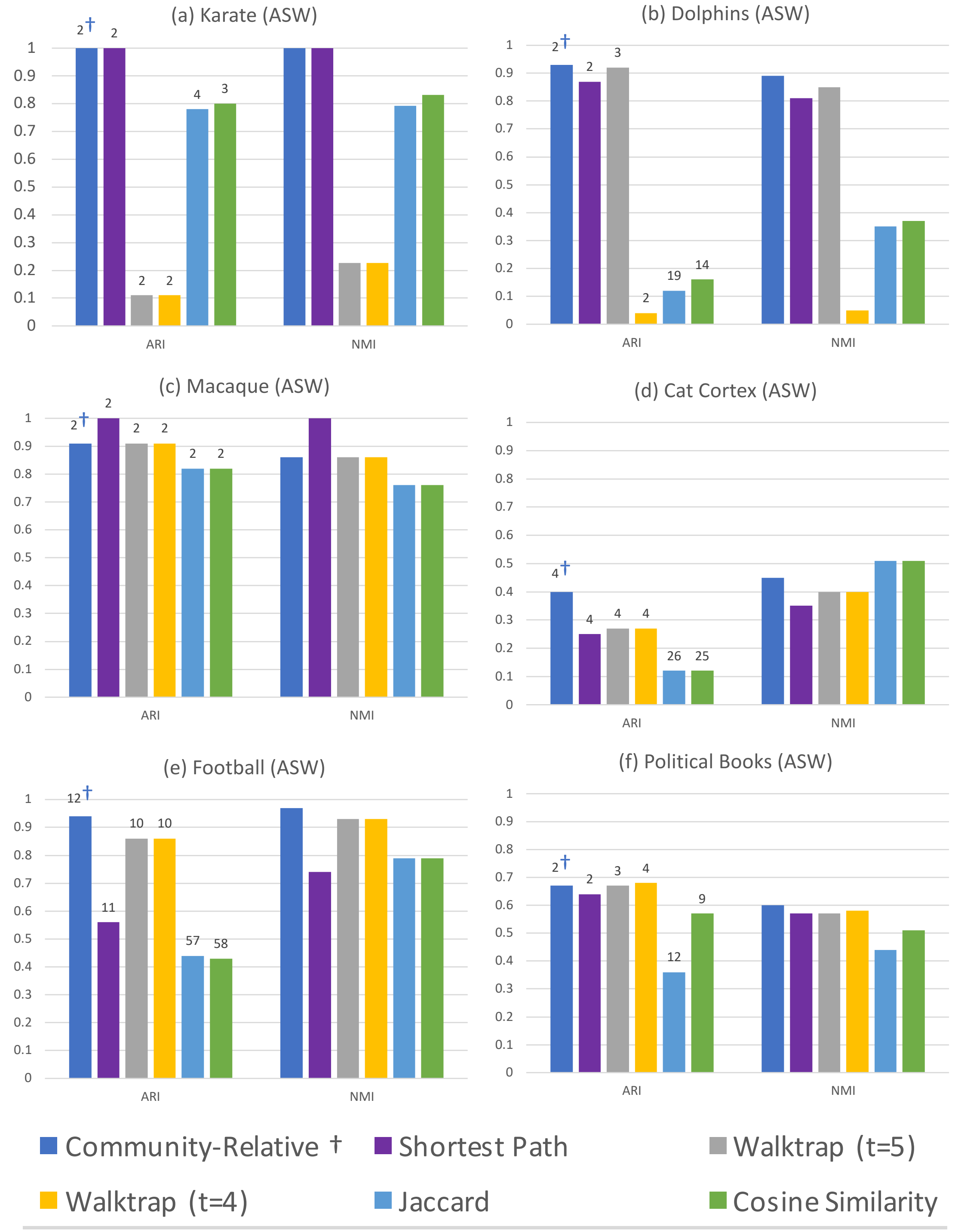

Figure 4: (a-f) ARI and NMI values for agglomerative clustering (employing average-linkage and an ASW stopping condition) for some common networks possessing reasonable ground truths, via a range of common distance measures.

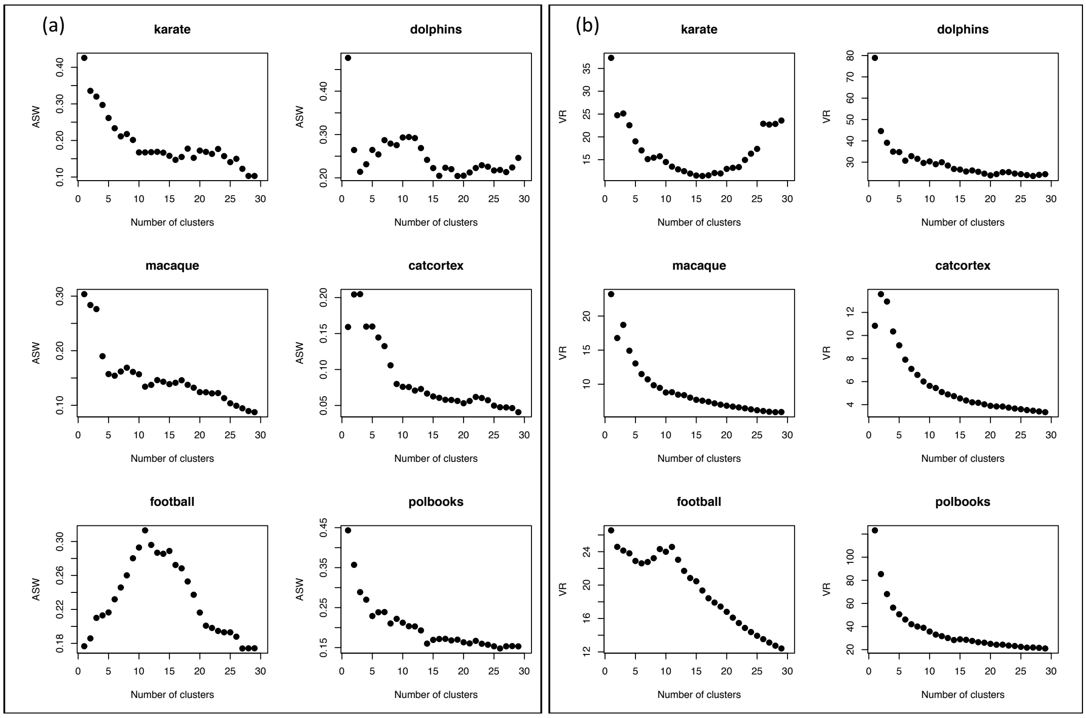

Figure 5: Plots of (a) ASW and (b) VR values versus number of clusters for some common networks possessing reasonable ground truths.

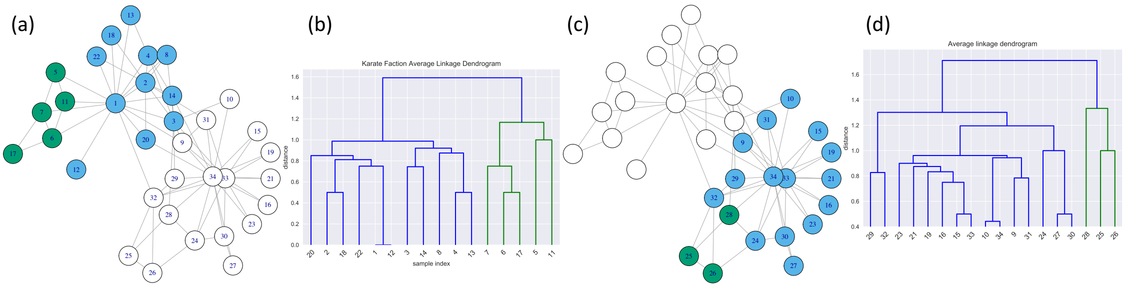

Figure 6: Clustering of the two factions of the karate network reported in [1].

	1	2	3	4	5	6	7	8	11	12	13	14	17	18	20	22
**1**	0.00	0.87	0.81	0.83	0.67	1.00	1.00	0.75	0.67	0.00	0.50	0.77	1.00	0.50	0.61	0.50
**2**	0.87	0.00	0.87	1.00	1.67	1.87	1.87	0.75	1.67	1.00	1.00	0.78	2.00	0.50	0.64	0.50
**3**	0.81	0.87	0.00	0.94	1.67	1.81	1.81	0.75	1.67	1.00	1.00	0.74	2.00	1.00	0.91	1.00
**4**	0.83	1.00	0.94	0.00	1.67	1.83	1.83	0.75	1.67	1.00	0.50	0.83	2.00	1.00	1.05	1.00
**5**	0.67	1.67	1.67	1.67	0.00	1.00	1.00	1.67	1.00	1.00	1.50	1.67	1.50	1.50	1.61	1.50
**6**	1.00	1.87	1.81	1.83	1.00	0.00	1.00	1.75	1.00	1.00	1.50	1.77	0.50	1.50	1.61	1.50
**7**	1.00	1.87	1.81	1.83	1.00	1.00	0.00	1.75	1.00	1.00	1.50	1.77	0.50	1.50	1.61	1.50
**8**	0.75	0.75	0.75	0.75	1.67	1.75	1.75	0.00	1.67	1.00	1.00	1.00	2.00	1.00	1.11	1.00
**11**	0.67	1.67	1.67	1.67	1.00	1.00	1.00	1.67	0.00	1.00	1.50	1.67	1.50	1.50	1.61	1.50
**12**	0.00	1.00	1.00	1.00	1.00	1.00	1.00	1.00	1.00	0.00	1.00	1.00	2.00	1.00	1.00	1.00
**13**	0.50	1.00	1.00	0.50	1.50	1.50	1.50	1.00	1.50	1.00	0.00	1.00	2.00	1.50	1.50	1.50
**14**	0.77	0.78	0.74	0.83	1.67	1.77	1.77	1.00	1.67	1.00	1.00	0.00	2.00	1.00	1.00	1.00
**17**	1.00	2.00	2.00	2.00	1.50	0.50	0.50	2.00	1.50	2.00	2.00	2.00	0.00	2.00	2.00	2.00
**18**	0.50	0.50	1.00	1.00	1.50	1.50	1.50	1.00	1.50	1.00	1.50	1.00	2.00	0.00	1.00	1.00
**20**	0.61	0.64	0.91	1.05	1.61	1.61	1.61	1.11	1.61	1.00	1.50	1.00	2.00	1.00	0.00	1.00
**22**	0.50	0.50	1.00	1.00	1.50	1.50	1.50	1.00	1.50	1.00	1.50	1.00	2.00	1.00	1.00	0.00

Figure 7: Community-relative distance matrix, $\boldsymbol{D}^*$, for the faction of the karate club network [1] depicted in Fig S6a

	23	24	25	26	27	28	29	30	31	32	33	34	35	36	37	38	39	40
**23**	0.00	1.14	1.00	1.00	1.00	1.00	1.00	1.46	2.28	2.32	1.50	1.66	1.43	1.50	0.78	1.36	0.88	0.74
**24**	1.14	0.00	1.37	1.37	1.37	1.37	1.37	1.30	2.10	2.17	1.44	1.30	1.27	1.37	1.20	1.24	1.14	0.44
**25**	1.00	1.37	0.00	1.00	1.00	1.00	1.00	1.00	2.00	2.00	1.50	1.50	1.50	1.00	1.00	1.00	0.50	0.50
**26**	1.00	1.37	1.00	0.00	1.00	1.00	1.00	1.00	2.00	2.00	1.50	1.50	1.50	1.00	1.00	1.00	0.50	0.50
**27**	1.00	1.37	1.00	1.00	0.00	1.00	1.00	1.00	2.00	2.00	1.50	1.50	1.50	1.00	1.00	1.00	0.50	0.50
**28**	1.00	1.37	1.00	1.00	1.00	0.00	1.00	1.00	2.00	2.00	1.50	1.50	1.50	1.00	1.00	1.00	0.50	0.50
**29**	1.00	1.37	1.00	1.00	1.00	1.00	0.00	1.00	2.00	2.00	1.50	1.50	1.50	1.00	1.00	1.00	0.50	0.50
**30**	1.46	1.30	1.00	1.00	1.00	1.00	1.00	0.00	1.33	1.33	1.00	1.15	1.54	1.00	1.41	1.40	1.20	1.00
**31**	2.28	2.10	2.00	2.00	2.00	2.00	2.00	1.33	0.00	1.00	2.33	1.33	1.67	2.00	2.32	1.00	1.67	1.33
**32**	2.32	2.17	2.00	2.00	2.00	2.00	2.00	1.33	1.00	0.00	2.00	1.33	1.67	2.00	2.33	1.00	1.33	1.33
**33**	1.50	1.44	1.50	1.50	1.50	1.50	1.50	1.00	2.33	2.00	0.00	1.50	1.50	0.50	1.50	1.50	1.00	0.50
**34**	1.66	1.30	1.50	1.50	1.50	1.50	1.50	1.15	1.33	1.33	1.50	0.00	1.54	1.50	1.66	1.37	1.32	0.97
**35**	1.43	1.27	1.50	1.50	1.50	1.50	1.50	1.54	1.67	1.67	1.50	1.54	0.00	1.58	1.47	0.83	1.09	0.63
**36**	1.50	1.37	1.00	1.00	1.00	1.00	1.00	1.00	2.00	2.00	0.50	1.50	1.58	0.00	1.43	1.50	1.00	0.75
**37**	0.78	1.20	1.00	1.00	1.00	1.00	1.00	1.41	2.32	2.33	1.50	1.66	1.47	1.43	0.00	1.36	0.78	0.70
**38**	1.36	1.24	1.00	1.00	1.00	1.00	1.00	1.40	1.00	1.00	1.50	1.37	0.83	1.50	1.36	0.00	1.35	1.13
**39**	0.88	1.14	0.50	0.50	0.50	0.50	0.50	1.20	1.67	1.33	1.00	1.32	1.09	1.00	0.78	1.35	0.00	0.91
**40**	0.74	0.44	0.50	0.50	0.50	0.50	0.50	1.00	1.33	1.33	0.50	0.97	0.63	0.75	0.70	1.13	0.91	0.00

Figure 8: Community-relative distance matrix, $\boldsymbol{D}^*$, for the faction of the karate club network [1] depicted in Fig S6c.

1	2	3	4	5	6	7	8	9	10	11	12	13	14	15	16	17	18	19	20	21	22	23	24	25	26	27	28	29	30	31	32	33	34
0.0	1.0	1.3	0.8	0.7	1.0	1.0	0.8	1.4	1.5	0.7	0.0	0.5	1.0	2.0	2.0	1.0	0.5	2.0	1.0	2.0	0.5	2.0	2.2	1.7	2.0	2.5	2.0	1.3	2.5	1.5	1.7	2.3	2.2
1.0	0.0	1.3	1.0	1.7	2.0	2.0	0.8	1.4	1.5	1.7	1.0	1.0	1.0	2.0	2.0	2.0	0.5	2.0	1.0	2.0	0.5	2.0	2.4	2.3	2.7	2.5	2.1	1.7	2.5	1.5	1.9	2.0	2.0
1.3	1.3	0.0	1.0	1.7	2.0	2.0	0.8	1.2	1.0	1.7	1.0	1.0	1.0	1.5	1.5	2.0	1.0	1.5	1.3	1.5	1.0	1.5	1.8	2.0	2.0	2.0	1.5	1.3	2.0	1.3	1.7	1.7	1.5
0.8	1.0	1.0	0.0	1.7	1.8	1.8	0.8	1.6	1.5	1.7	1.0	0.5	1.0	2.0	2.0	2.0	1.0	2.0	1.3	2.0	1.0	2.0	2.4	2.3	2.7	2.5	2.0	1.7	2.5	1.8	1.8	2.0	2.2
0.7	1.7	1.7	1.7	0.0	1.0	1.0	1.7	1.7	2.5	1.0	1.0	1.5	1.7	3.0	3.0	1.5	1.5	3.0	1.7	3.0	1.5	3.0	3.2	2.7	2.7	3.5	2.7	2.3	3.5	2.5	1.7	2.7	2.7
1.0	2.0	2.0	1.8	1.0	0.0	1.0	1.8	2.0	2.5	1.0	1.0	1.5	2.0	3.0	3.0	0.5	1.5	3.0	2.0	3.0	1.5	3.0	3.2	2.7	3.0	3.5	3.0	2.3	3.5	2.5	2.0	3.0	3.0
1.0	2.0	2.0	1.8	1.0	1.0	0.0	1.8	2.0	2.5	1.0	1.0	1.5	2.0	3.0	3.0	0.5	1.5	3.0	2.0	3.0	1.5	3.0	3.2	2.7	3.0	3.5	3.0	2.3	3.5	2.5	2.0	3.0	3.0
0.8	0.8	0.8	0.8	1.7	1.8	1.8	0.0	1.5	1.8	1.7	1.0	1.0	1.0	2.5	2.5	2.0	1.0	2.5	1.5	2.5	1.0	2.5	2.6	2.3	2.7	3.0	1.8	1.8	2.8	1.8	1.8	1.8	2.0
1.4	1.4	1.2	1.6	1.7	2.0	2.0	1.5	0.0	1.0	1.7	1.0	1.5	1.4	1.0	1.0	2.0	1.5	1.0	1.3	1.0	1.5	1.0	1.8	2.2	2.3	1.5	1.6	1.3	1.5	1.0	1.4	1.0	1.2
1.5	1.5	1.0	1.5	2.5	2.5	2.5	1.8	1.0	0.0	2.5	2.0	2.0	1.0	1.5	1.5	3.0	2.0	1.5	1.5	1.5	2.0	1.5	1.5	2.0	2.3	1.5	1.0	1.0	1.5	1.5	1.5	1.0	1.0
0.7	1.7	1.7	1.7	1.0	1.0	1.0	1.7	1.7	2.5	0.0	1.0	1.5	1.7	3.0	3.0	1.5	1.5	3.0	1.7	3.0	1.5	3.0	3.2	2.7	2.7	3.5	2.7	2.3	3.5	2.5	1.7	2.7	2.7
0.0	1.0	1.0	1.0	1.0	1.0	1.0	1.0	1.0	2.0	1.0	0.0	1.0	1.0	3.0	3.0	2.0	1.0	3.0	1.0	3.0	1.0	3.0	3.0	2.0	2.0	3.0	2.0	2.0	3.0	2.0	1.0	2.0	2.0
0.5	1.0	1.0	0.5	1.5	1.5	1.5	1.0	1.5	2.0	1.5	1.0	0.0	1.0	3.0	3.0	2.0	1.5	3.0	1.5	3.0	1.5	3.0	3.0	2.5	2.5	3.0	2.0	2.0	3.0	2.0	1.5	2.0	2.0
1.0	1.0	1.0	1.0	1.7	2.0	2.0	1.0	1.4	1.0	1.7	1.0	1.0	0.0	1.5	1.5	2.0	1.0	1.5	1.0	1.5	1.0	1.5	2.0	2.3	2.3	1.5	1.6	1.3	1.8	1.5	1.6	1.6	1.6
2.0	2.0	1.5	2.0	3.0	3.0	3.0	2.5	1.0	1.5	3.0	3.0	3.0	1.5	0.0	1.0	4.0	3.0	1.0	1.5	1.0	3.0	1.0	1.0	2.0	2.0	1.5	1.5	1.5	1.0	1.0	1.0	0.5	0.5
2.0	2.0	1.5	2.0	3.0	3.0	3.0	2.5	1.0	1.5	3.0	3.0	3.0	1.5	1.0	0.0	4.0	3.0	1.0	1.5	1.0	3.0	1.0	1.0	2.0	2.0	1.5	1.5	1.5	1.0	1.0	1.0	0.5	0.5
1.0	2.0	2.0	2.0	1.5	0.5	0.5	2.0	2.0	3.0	1.5	2.0	2.0	2.0	4.0	4.0	0.0	2.0	4.0	2.0	4.0	2.0	4.0	4.0	3.0	3.0	4.0	3.0	3.0	4.0	3.0	2.0	3.0	3.0
0.5	0.5	1.0	1.0	1.5	1.5	1.5	1.0	1.5	2.0	1.5	1.0	1.5	1.0	3.0	3.0	2.0	0.0	3.0	1.0	3.0	1.0	3.0	3.0	2.5	2.5	3.0	2.0	2.0	3.0	1.5	1.5	2.0	2.0
2.0	2.0	1.5	2.0	3.0	3.0	3.0	2.5	1.0	1.5	3.0	3.0	3.0	1.5	1.0	1.0	4.0	3.0	0.0	1.5	1.0	3.0	1.0	1.0	2.0	2.0	1.5	1.5	1.5	1.0	1.0	1.0	0.5	0.5
1.0	1.0	1.3	1.3	1.7	2.0	2.0	1.5	1.3	1.5	1.7	1.0	1.5	1.0	1.5	1.5	2.0	1.0	1.5	0.0	1.5	1.0	1.5	2.0	2.3	2.3	1.5	1.7	1.7	1.8	1.3	1.3	1.7	1.3
2.0	2.0	1.5	2.0	3.0	3.0	3.0	2.5	1.0	1.5	3.0	3.0	3.0	1.5	1.0	1.0	4.0	3.0	1.0	1.5	0.0	3.0	1.0	1.0	2.0	2.0	1.5	1.5	1.5	1.0	1.0	1.0	0.5	0.5
0.5	0.5	1.0	1.0	1.5	1.5	1.5	1.0	1.5	2.0	1.5	1.0	1.5	1.0	3.0	3.0	2.0	1.0	3.0	1.0	3.0	0.0	3.0	3.0	2.5	2.5	3.0	2.0	2.0	3.0	1.5	1.5	2.0	2.0
2.0	2.0	1.5	2.0	3.0	3.0	3.0	2.5	1.0	1.5	3.0	3.0	3.0	1.5	1.0	1.0	4.0	3.0	1.0	1.5	1.0	3.0	0.0	1.0	2.0	2.0	1.5	1.5	1.5	1.0	1.0	1.0	0.5	0.5
2.2	2.4	1.8	2.4	3.2	3.2	3.2	2.6	1.8	1.5	3.2	3.0	3.0	2.0	1.0	1.0	4.0	3.0	1.0	2.0	1.0	3.0	1.0	0.0	1.3	1.3	1.0	1.3	1.7	1.0	1.8	1.4	1.2	1.0
1.7	2.3	2.0	2.3	2.7	2.7	2.7	2.3	2.2	2.0	2.7	2.0	2.5	2.3	2.0	2.0	3.0	2.5	2.0	2.3	2.0	2.5	2.0	1.3	0.0	1.0	2.3	1.3	1.7	2.0	2.3	1.0	1.7	1.3
2.0	2.7	2.0	2.7	2.7	3.0	3.0	2.7	2.3	2.3	2.7	2.0	2.5	2.3	2.0	2.0	3.0	2.5	2.0	2.3	2.0	2.5	2.0	1.3	1.0	0.0	2.0	1.3	1.7	2.0	2.3	1.0	1.3	1.3
2.5	2.5	2.0	2.5	3.5	3.5	3.5	3.0	1.5	1.5	3.5	3.0	3.0	1.5	1.5	1.5	4.0	3.0	1.5	1.5	1.5	3.0	1.5	1.0	2.3	2.0	0.0	1.5	1.5	0.5	1.5	1.5	1.0	0.5
2.0	2.1	1.5	2.0	2.7	3.0	3.0	1.8	1.6	1.0	2.7	2.0	2.0	1.6	1.5	1.5	3.0	2.0	1.5	1.7	1.5	2.0	1.5	1.3	1.3	1.3	1.5	0.0	1.3	1.5	1.8	1.5	1.3	1.3
1.3	1.7	1.3	1.7	2.3	2.3	2.3	1.8	1.3	1.0	2.3	2.0	2.0	1.3	1.5	1.5	3.0	2.0	1.5	1.7	1.5	2.0	1.5	1.7	1.7	1.7	1.5	1.3	0.0	1.7	1.7	1.0	1.0	1.0
2.5	2.5	2.0	2.5	3.5	3.5	3.5	2.8	1.5	1.5	3.5	3.0	3.0	1.8	1.0	1.0	4.0	3.0	1.0	1.8	1.0	3.0	1.0	1.0	2.0	2.0	0.5	1.5	1.7	0.0	1.5	1.5	1.0	0.8
1.5	1.5	1.3	1.8	2.5	2.5	2.5	1.8	1.0	1.5	2.5	2.0	2.0	1.5	1.0	1.0	3.0	1.5	1.0	1.3	1.0	1.5	1.0	1.8	2.3	2.3	1.5	1.8	1.7	1.5	0.0	1.5	1.0	1.0
1.7	1.9	1.7	1.8	1.7	2.0	2.0	1.8	1.4	1.5	1.7	1.0	1.5	1.6	1.0	1.0	2.0	1.5	1.0	1.3	1.0	1.5	1.0	1.4	1.0	1.0	1.5	1.5	1.0	1.5	1.5	0.0	1.5	1.3
2.3	2.0	1.7	2.0	2.7	3.0	3.0	1.8	1.0	1.0	2.7	2.0	2.0	1.6	0.5	0.5	3.0	2.0	0.5	1.7	0.5	2.0	0.5	1.2	1.7	1.3	1.0	1.3	1.0	1.0	1.0	1.5	0.0	1.0
2.2	2.0	1.5	2.2	2.7	3.0	3.0	2.0	1.2	1.0	2.7	2.0	2.0	1.6	0.5	0.5	3.0	2.0	0.5	1.3	0.5	2.0	0.5	1.0	1.3	1.3	0.5	1.3	1.0	0.8	1.0	1.3	1.0	0.0

Figure 9: Community-relative distance matrix, $\boldsymbol{D}^*$, for all node pairs in the karate club network [1].

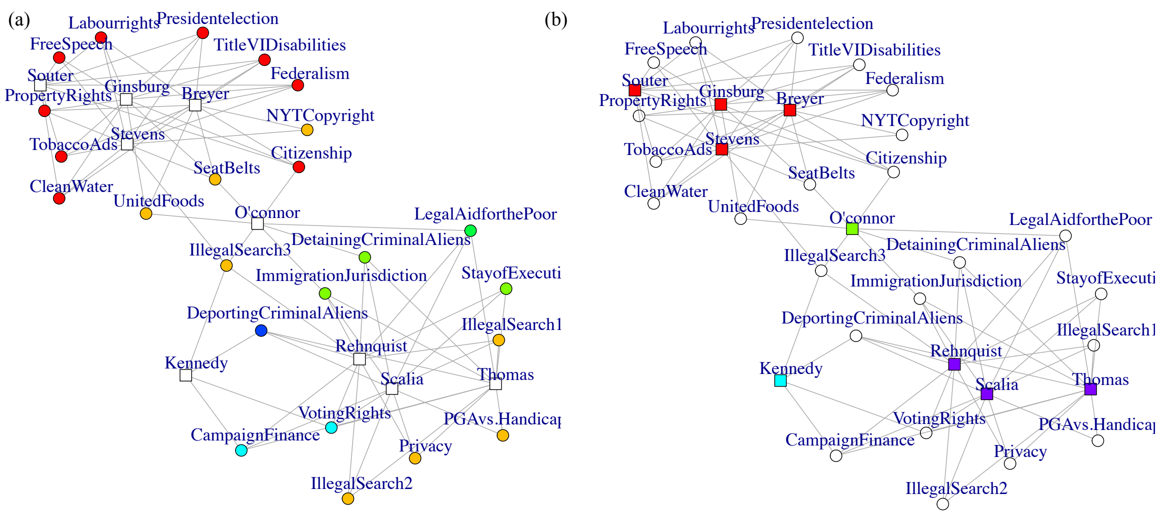

Figure 10: (a) Seven-clustering (via community relative distances and average-linkage clustering) for the nodes representing the 24 cases from the US Supreme Court network [2]. Note the two cases with unanimous decisions for those voting have been excluded. (b) Four-clustering for the nodes representing the nine justices in the network [2].

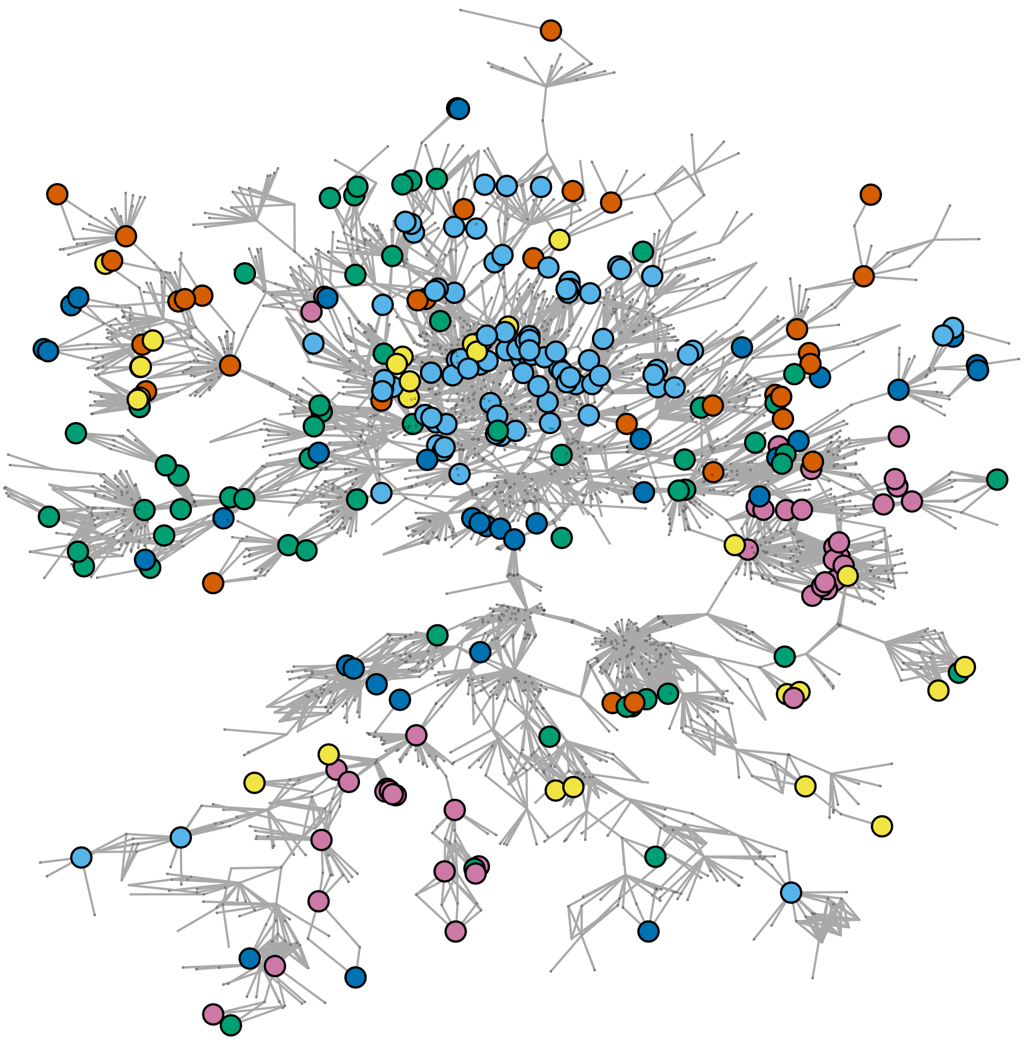

Figure 11: The bipartite Human Disease Network [3]. Cancer, Neurological, Skeletal, Hematological, Metabolic and Ophthalmological nodes are coded in blue, green, yellow, orange, dark blue and pink, respectively.

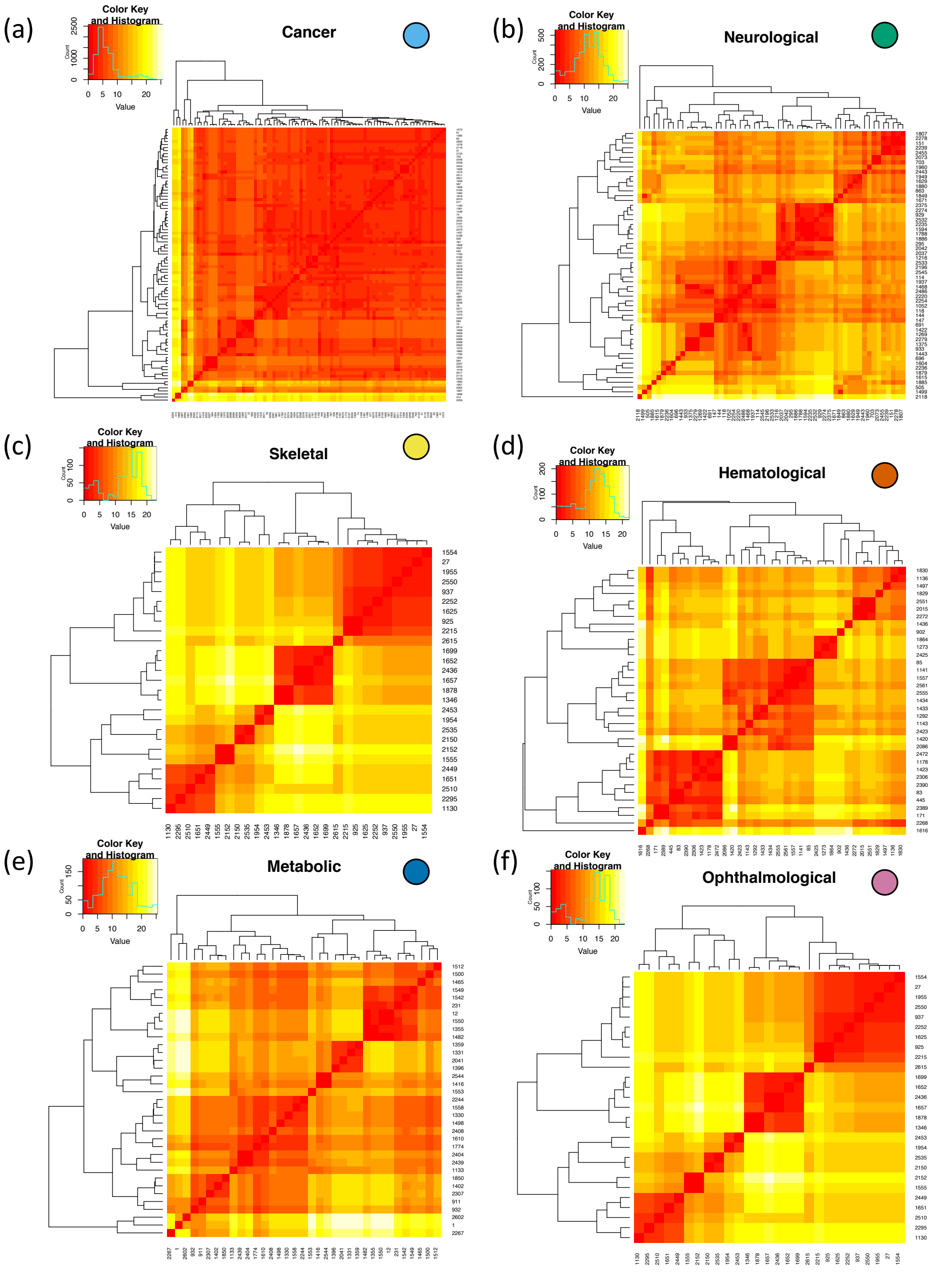

Figure 12: Community-relative distances for several diseases from the bipartite human disease Network [3] (as heat maps). Associated dendrograms indicate average-linkage clustering results. Colors at the top-right correspond to those employed in Figure S11.

V1	V2	V3	V3A	V4	V4t	VOT	VP	MT	MST d/p	MSTl	PO	LIP	PIP	VIP	DP	7a	FST	PITd	PITv	CITd	CITv	AITd	AITv	STPp	STPa	TF	TH	FEF	46	3a	3b	1	2	5	Ri	SII	7b	4	6	SMA	Ig	Id	35	3[illegible]
0.0	0.9	0.9	1.1	1.0	1.1	1.6	1.3	0.9	1.3	1.4	1.0	1.4	1.1	1.5	1.5	1.8	1.3	1.9	1.9	2.0	2.0	2.0	1.9	2.1	2.4	1.8	1.9	1.3	1.8	2.5	3.0	2.5	2.4	2.3	3.1	2.4	2.1	2.1	2.0	2.1	3.0	3.1	2.6	2.[illegible]
0.9	0.0	1.1	1.1	1.3	0.9	1.2	1.1	1.0	1.3	1.2	1.1	1.3	1.1	1.3	1.3	1.6	1.2	1.5	1.7	1.8	1.9	2.0	1.9	1.7	2.0	1.7	1.9	1.2	1.7	2.0	2.0	2.0	2.0	2.0	2.3	2.2	2.1	1.8	1.8	1.8	2.5	2.5	2.5	2.[illegible]
0.9	1.1	0.0	1.2	1.2	1.0	1.6	1.1	1.0	1.1	1.2	1.1	1.2	1.2	1.4	1.3	1.4	1.1	1.6	1.6	1.8	1.8	1.8	1.7	1.6	1.8	1.6	1.8	1.2	1.5	2.0	2.0	2.0	2.0	1.8	2.3	2.2	2.0	1.8	1.7	1.6	2.3	2.3	2.3	2.[illegible]
1.1	1.1	1.2	0.0	1.2	1.0	1.4	1.2	1.0	1.2	1.2	1.1	1.1	1.1	1.2	1.2	1.5	1.1	1.6	1.7	1.8	1.9	2.0	1.9	1.7	2.0	1.5	1.8	1.1	1.5	2.2	3.0	2.2	2.2	2.0	2.5	2.1	2.0	2.0	1.9	1.9	2.7	2.8	2.3	2.[illegible]
1.0	1.3	1.2	1.2	0.0	1.3	0.8	1.2	1.3	1.4	1.4	1.3	1.5	1.1	1.8	1.3	1.6	1.3	1.1	1.1	1.2	1.1	1.3	0.9	1.4	1.4	1.4	1.3	1.4	1.6	2.5	2.8	2.4	2.3	2.1	2.3	2.4	2.2	2.1	2.1	2.0	2.3	2.3	2.0	1.[illegible]
1.1	0.9	1.0	1.0	1.3	0.0	1.6	1.3	0.9	1.2	1.3	1.1	1.2	1.3	1.2	1.4	1.6	1.1	1.6	1.7	1.8	1.9	2.0	1.9	1.7	2.0	1.6	1.8	1.1	1.6	2.2	3.0	2.2	2.2	2.1	3.0	2.1	2.1	2.0	2.0	2.0	3.0	2.8	2.4	2.[illegible]
1.6	1.2	1.6	1.4	0.8	1.6	0.0	1.2	1.4	1.6	1.8	1.6	1.4	1.4	1.6	1.6	2.0	1.0	1.4	1.4	1.6	1.4	1.6	1.4	2.0	2.2	1.4	1.6	1.2	1.4	2.6	3.0	2.6	2.6	2.2	3.0	2.0	2.0	2.0	2.0	2.2	3.0	3.0	2.2	2.[illegible]
1.3	1.1	1.1	1.2	1.2	1.3	1.2	0.0	1.1	1.1	1.2	1.2	1.1	1.2	1.4	1.2	1.4	1.1	1.5	1.5	1.8	1.8	1.8	1.7	1.6	1.8	1.6	1.8	1.2	1.4	2.0	2.0	2.0	2.0	1.8	2.3	2.1	1.9	1.8	1.7	1.6	2.3	2.3	2.3	2.[illegible]
0.9	1.0	1.0	1.0	1.3	0.9	1.4	1.1	0.0	1.2	1.1	1.1	1.2	1.1	1.3	1.1	1.5	1.2	1.5	1.6	1.8	1.9	1.8	1.9	1.5	1.8	1.6	1.8	1.1	1.6	2.0	2.0	2.0	2.0	1.9	2.3	2.1	1.9	1.7	1.7	1.6	2.3	2.3	2.3	2.[illegible]
1.3	1.3	1.1	1.2	1.4	1.2	1.6	1.1	1.2	0.0	1.0	1.1	1.1	1.2	1.2	1.2	1.3	1.1	1.6	1.6	1.8	1.8	1.6	1.9	1.4	1.4	1.5	1.7	1.1	1.4	2.0	2.0	2.0	1.8	1.7	2.3	2.0	1.8	1.6	1.5	1.5	2.3	2.3	2.0	1.[illegible]
1.4	1.2	1.2	1.2	1.4	1.3	1.8	1.2	1.1	1.0	0.0	1.1	1.2	1.6	1.3	1.4	1.5	1.0	1.8	1.7	2.0	2.0	1.9	2.0	1.6	1.8	1.7	1.8	0.9	1.5	2.0	2.0	2.0	2.0	1.9	2.3	2.1	1.9	1.8	1.7	1.8	2.5	2.5	2.4	1.[illegible]
1.0	1.1	1.1	1.1	1.3	1.1	1.6	1.2	1.1	1.1	1.1	0.0	1.2	1.0	1.3	1.2	1.5	1.1	1.9	1.9	2.1	2.1	1.9	2.1	1.9	2.0	1.7	1.9	1.1	1.5	2.0	2.0	2.0	1.9	1.9	2.3	2.2	1.9	1.8	1.6	1.6	2.5	2.5	2.4	2.[illegible]
1.4	1.3	1.2	1.1	1.5	1.2	1.4	1.1	1.2	1.1	1.2	1.2	0.0	1.2	1.2	1.0	1.2	1.2	1.5	1.4	1.7	1.6	1.5	1.6	1.4	1.6	1.4	1.6	1.2	1.4	2.0	2.0	1.6	1.5	1.4	1.5	1.8	1.4	1.3	1.3	1.1	2.0	2.0	1.8	1.[illegible]
1.1	1.1	1.2	1.1	1.1	1.3	1.4	1.2	1.1	1.2	1.6	1.0	1.2	0.0	1.3	1.3	1.6	1.2	1.9	1.9	2.0	2.0	2.0	1.9	2.1	2.2	1.6	1.8	1.2	1.6	2.3	2.8	2.3	2.1	2.1	2.8	2.1	2.0	2.1	2.0	2.1	3.0	3.0	2.3	2.[illegible]
1.5	1.3	1.4	1.2	1.8	1.2	1.6	1.4	1.3	1.2	1.3	1.3	1.2	1.3	0.0	1.3	1.3	1.4	1.9	1.9	2.2	2.1	1.9	2.3	1.7	1.8	1.6	2.0	1.3	1.5	1.0	1.0	1.0	1.0	1.1	1.3	1.2	1.1	0.9	0.9	0.9	1.7	1.8	1.8	1.[illegible]
1.5	1.3	1.3	1.2	1.3	1.4	1.6	1.2	1.1	1.2	1.4	1.2	1.0	1.3	1.3	0.0	1.3	1.2	1.5	1.6	1.8	1.9	1.7	1.9	1.5	1.8	1.4	1.7	1.1	1.4	2.3	2.5	2.3	2.1	2.0	2.3	2.1	1.8	1.9	1.8	1.8	2.3	2.3	2.3	1.[illegible]
1.8	1.6	1.4	1.5	1.6	1.6	2.0	1.4	1.5	1.3	1.5	1.5	1.2	1.6	1.3	1.3	0.0	1.4	1.6	1.5	1.7	1.6	1.4	1.7	1.4	1.2	1.5	1.6	1.3	1.3	1.7	1.8	1.5	1.5	1.5	1.8	1.7	1.5	1.4	1.4	1.3	2.0	2.0	1.8	1.[illegible]
1.3	1.2	1.1	1.1	1.3	1.1	1.0	1.1	1.2	1.1	1.0	1.1	1.2	1.2	1.4	1.2	1.4	0.0	1.5	1.4	1.3	1.3	1.4	1.3	1.3	1.4	1.4	1.5	1.1	1.3	2.0	2.0	2.0	1.9	1.8	2.3	2.1	1.9	1.8	1.6	1.6	2.3	2.3	2.2	1.[illegible]
1.9	1.5	1.6	1.6	1.1	1.6	1.4	1.5	1.5	1.6	1.8	1.9	1.5	1.9	1.9	1.5	1.6	1.5	0.0	1.0	1.5	1.4	1.4	1.4	1.6	1.6	1.3	1.3	1.4	1.4	2.5	2.8	2.5	2.5	2.1	2.5	2.3	2.3	2.1	2.3	2.3	2.3	2.3	2.0	1.[illegible]
1.9	1.7	1.6	1.7	1.1	1.7	1.4	1.5	1.6	1.6	1.7	1.9	1.4	1.9	1.9	1.6	1.5	1.4	1.0	0.0	1.0	1.1	1.2	1.0	1.3	1.2	1.3	1.3	1.5	1.3	2.5	2.8	2.4	2.3	2.1	2.3	2.3	2.2	2.0	2.0	2.0	2.3	2.3	2.0	1.[illegible]
2.0	1.8	1.8	1.8	1.2	1.8	1.6	1.8	1.8	1.8	2.0	2.1	1.7	2.0	2.2	1.8	1.7	1.3	1.5	1.0	0.0	1.0	1.3	1.2	1.5	1.4	1.2	0.8	1.5	1.2	3.2	3.2	3.0	2.7	2.2	3.0	2.2	2.2	2.2	2.2	2.5	2.8	2.8	2.0	1.[illegible]
2.0	1.9	1.8	1.9	1.1	1.9	1.4	1.8	1.9	1.8	2.0	2.1	1.6	2.0	2.1	1.9	1.6	1.3	1.4	1.1	1.0	0.0	1.3	1.0	1.5	1.2	1.1	1.1	1.5	1.1	3.1	3.1	2.9	2.6	2.1	2.8	2.1	2.1	2.1	2.1	2.5	2.7	2.5	1.9	1.[illegible]
2.0	2.0	1.8	2.0	1.3	2.0	1.6	1.8	1.8	1.6	1.9	1.9	1.5	2.0	1.9	1.7	1.4	1.4	1.4	1.2	1.3	1.3	0.0	1.1	1.3	1.0	1.3	1.2	1.4	1.1	2.3	2.5	2.3	2.3	2.2	2.3	2.1	2.0	2.0	2.0	2.0	2.3	2.3	1.9	1.[illegible]
1.9	1.9	1.7	1.9	0.9	1.9	1.4	1.7	1.9	1.9	2.0	2.1	1.6	1.9	2.3	1.9	1.7	1.3	1.4	1.0	1.2	1.0	1.1	0.0	1.4	1.6	1.3	1.1	1.7	1.3	3.2	3.3	3.0	2.7	2.3	2.8	2.3	2.3	2.3	2.3	2.6	2.7	2.5	2.0	1.[illegible]
2.1	1.7	1.6	1.7	1.4	1.7	2.0	1.6	1.5	1.4	1.6	1.9	1.4	2.1	1.7	1.5	1.4	1.3	1.6	1.3	1.5	1.5	1.3	1.4	0.0	1.0	1.3	1.3	1.4	1.3	2.5	2.8	2.4	2.3	2.1	2.3	2.1	2.1	2.0	2.1	2.0	2.3	2.3	2.0	1.[illegible]
2.4	2.0	1.8	2.0	1.4	2.0	2.2	1.8	1.8	1.4	1.8	2.0	1.6	2.2	1.8	1.8	1.2	1.4	1.6	1.2	1.4	1.2	1.0	1.6	1.0	0.0	1.0	1.0	1.4	0.8	2.5	2.8	2.5	2.2	2.0	2.3	1.8	1.8	1.8	2.0	2.3	2.3	2.3	1.6	1.[illegible]
1.8	1.7	1.6	1.5	1.4	1.6	1.4	1.6	1.6	1.5	1.7	1.7	1.4	1.6	1.6	1.4	1.5	1.4	1.3	1.3	1.2	1.1	1.3	1.3	1.3	1.0	0.0	1.1	1.4	1.3	2.2	2.3	2.1	2.0	1.7	1.8	2.0	1.7	1.9	1.7	1.6	1.7	1.5	1.3	1.[illegible]
1.9	1.9	1.8	1.8	1.3	1.8	1.6	1.8	1.8	1.7	1.8	1.9	1.6	1.8	2.0	1.7	1.6	1.5	1.3	1.3	0.8	1.1	1.2	1.1	1.3	1.0	1.1	0.0	1.4	1.3	2.3	2.5	2.4	2.4	2.1	2.3	2.2	2.1	2.2	2.2	2.1	2.0	1.8	1.8	1.[illegible]
1.3	1.2	1.2	1.1	1.4	1.1	1.2	1.2	1.1	1.1	0.9	1.1	1.2	1.2	1.3	1.1	1.3	1.1	1.4	1.5	1.5	1.5	1.4	1.7	1.4	1.4	1.4	1.4	0.0	1.3	1.8	2.0	1.9	1.7	1.7	2.0	1.8	1.6	1.5	1.5	1.4	2.0	1.8	1.8	1.[illegible]
1.8	1.7	1.5	1.5	1.6	1.6	1.4	1.4	1.6	1.4	1.5	1.5	1.4	1.6	1.5	1.4	1.3	1.3	1.4	1.3	1.2	1.1	1.1	1.3	1.3	0.8	1.3	1.3	1.3	0.0	1.5	1.8	1.6	1.5	1.5	1.5	1.6	1.5	1.4	1.4	1.4	1.3	1.3	1.3	1.[illegible]
2.5	2.0	2.0	2.2	2.5	2.2	2.6	2.0	2.0	2.0	2.0	2.0	2.0	2.3	1.0	2.3	1.7	2.0	2.5	2.5	3.2	3.1	2.3	3.2	2.5	2.5	2.2	2.3	1.8	1.5	0.0	0.8	1.0	0.8	1.2	1.8	0.8	1.2	1.2	1.2	1.5	1.8	1.8	2.3	2.[illegible]
3.0	2.0	2.0	3.0	2.8	3.0	3.0	2.0	2.0	2.0	2.0	2.0	2.0	2.8	1.0	2.5	1.8	2.0	2.8	2.8	3.2	3.1	2.5	3.3	2.8	2.8	2.3	2.5	2.0	1.8	0.8	0.0	0.8	0.8	1.3	1.8	0.8	1.3	1.3	1.3	1.8	1.8	1.8	2.3	2.[illegible]
2.5	2.0	2.0	2.2	2.4	2.2	2.6	2.0	2.0	2.0	2.0	2.0	1.6	2.3	1.0	2.3	1.5	2.0	2.5	2.4	3.0	2.9	2.3	3.0	2.4	2.5	2.1	2.4	1.9	1.6	1.0	0.8	0.0	0.9	1.1	1.3	0.9	1.1	1.1	1.1	1.4	1.8	1.9	2.0	2.[illegible]
2.4	2.0	2.0	2.2	2.3	2.2	2.6	2.0	2.0	1.8	2.0	1.9	1.5	2.1	1.0	2.1	1.5	1.9	2.5	2.3	2.7	2.6	2.3	2.7	2.3	2.2	2.0	2.4	1.7	1.5	0.8	0.8	0.9	0.0	1.2	1.3	1.1	1.1	1.1	1.1	1.1	1.8	2.1	2.0	2.[illegible]
2.3	2.0	1.8	2.0	2.1	2.1	2.2	1.8	1.9	1.7	1.9	1.9	1.4	2.1	1.1	2.0	1.5	1.8	2.1	2.1	2.2	2.1	2.2	2.3	2.1	2.0	1.7	2.1	1.7	1.5	1.2	1.3	1.1	1.2	0.0	1.0	1.2	1.0	1.2	1.1	1.0	1.5	1.8	1.8	2.[illegible]
3.1	2.3	2.3	2.5	2.3	3.0	3.0	2.3	2.3	2.3	2.3	2.3	1.5	2.8	1.3	2.3	1.8	2.3	2.5	2.3	3.0	2.8	2.3	2.8	2.3	2.3	1.8	2.3	2.0	1.5	1.8	1.8	1.3	1.3	1.0	0.0	0.8	0.8	1.3	1.3	1.5	1.0	1.5	1.8	1.[illegible]
2.4	2.2	2.2	2.1	2.4	2.1	2.0	2.1	2.1	2.0	2.1	2.2	1.8	2.1	1.2	2.1	1.7	2.1	2.3	2.3	2.2	2.1	2.1	2.3	2.1	1.8	2.0	2.2	1.8	1.6	0.8	0.8	0.9	1.1	1.2	0.8	0.0	1.2	1.1	1.3	1.3	1.2	1.3	1.3	1.[illegible]
2.1	2.1	2.0	2.0	2.2	2.1	2.0	1.9	1.9	1.8	1.9	1.9	1.4	2.0	1.1	1.8	1.5	1.9	2.3	2.2	2.2	2.1	2.0	2.3	2.1	1.8	1.7	2.1	1.6	1.5	1.2	1.3	1.1	1.1	1.0	0.8	1.2	0.0	1.1	1.0	1.0	1.3	1.5	1.5	1.[illegible]
2.1	1.8	1.8	2.0	2.1	2.0	2.0	1.8	1.7	1.6	1.8	1.8	1.3	2.1	0.9	1.9	1.4	1.8	2.1	2.0	2.2	2.1	2.0	2.3	2.0	1.8	1.9	2.2	1.5	1.4	1.2	1.3	1.1	1.1	1.2	1.3	1.1	1.1	0.0	1.1	1.1	1.7	1.8	1.8	1.[illegible]
2.0	1.8	1.7	1.9	2.1	2.0	2.0	1.7	1.7	1.5	1.7	1.6	1.3	2.0	0.9	1.8	1.4	1.6	2.3	2.0	2.2	2.1	2.0	2.3	2.1	2.0	1.7	2.2	1.5	1.4	1.2	1.3	1.1	1.1	1.1	1.3	1.3	1.0	1.1	0.0	1.0	1.8	2.0	2.0	1.[illegible]
2.1	1.8	1.6	1.9	2.0	2.0	2.2	1.6	1.6	1.5	1.8	1.6	1.1	2.1	0.9	1.8	1.3	1.6	2.3	2.0	2.5	2.5	2.0	2.6	2.0	2.3	1.6	2.1	1.4	1.4	1.5	1.8	1.4	1.1	1.0	1.5	1.3	1.0	1.1	1.0	0.0	2.0	2.3	2.3	2.[illegible]
3.0	2.5	2.3	2.7	2.3	3.0	3.0	2.3	2.3	2.3	2.5	2.5	2.0	3.0	1.7	2.3	2.0	2.3	2.3	2.3	2.8	2.7	2.3	2.7	2.3	2.3	1.7	2.0	2.0	1.3	1.8	1.8	1.8	1.8	1.5	1.0	1.2	1.3	1.7	1.8	2.0	0.0	0.8	1.3	1.[illegible]
3.1	2.5	2.3	2.8	2.3	2.8	3.0	2.3	2.3	2.3	2.5	2.5	2.0	3.0	1.8	2.3	2.0	2.3	2.3	2.3	2.8	2.5	2.3	2.5	2.3	2.3	1.5	1.8	1.8	1.3	1.8	1.8	1.9	2.1	1.8	1.5	1.3	1.5	1.8	2.0	2.3	0.8	0.0	1.3	1.[illegible]
2.6	2.5	2.3	2.3	2.0	2.4	2.2	2.3	2.3	2.0	2.4	2.4	1.8	2.3	1.8	2.3	1.8	2.2	2.0	2.0	2.0	1.9	1.9	2.0	2.0	1.6	1.3	1.8	1.8	1.3	2.3	2.3	2.0	2.0	1.8	1.8	1.3	1.5	1.8	2.0	2.3	1.3	1.3	0.0	1.[illegible]
2.1	2.1	2.0	2.0	1.8	2.0	2.0	2.0	2.0	1.8	1.9	2.1	1.7	2.1	1.7	1.9	1.5	1.8	1.9	1.7	1.8	1.8	1.6	1.7	1.6	1.4	1.5	1.5	1.5	1.2	2.3	2.5	2.2	2.0	2.0	1.8	1.5	1.7	1.7	1.8	2.1	1.5	1.3	1.0	0.[illegible]

Figure 13: The community-relative distance matrix, $\boldsymbol{D}^*$, for the Macaque cortical network [4].

	V1	V2	V3	V3A	V4	V4t	VOT	VP	MT	MST d/p	MSTl	PO	LIP	PIP	VIP	DP	7a	FST	PITd	PITv	CITd	CITv	AITd	AITv	STPp	STPa	TF	TH	FEF	46	3a	3b	1	2	5	Ri	SII	7b	4	6	SMA	Ig	Id	35	36
V1	0	1	1	1	1	1	2	2	1	2	2	1	2	1	2	2	2	2	2	2	2	2	3	2	3	3	2	2	2	2	3	4	3	3	3	4	3	3	3	3	3	4	4	3	3
V2	1	0	1	1	1	1	1	1	1	1	1	1	2	1	1	2	2	1	2	2	2	2	2	2	2	3	2	2	1	2	2	3	2	2	2	3	2	2	2	2	2	3	3	3	2
V3	1	1	0	1	1	1	2	2	1	1	2	1	1	1	1	2	2	1	2	2	2	2	2	2	2	2	1	2	1	2	2	3	2	2	2	3	2	2	2	2	2	3	3	2	2
V3A	1	1	1	0	1	2	2	1	1	1	1	1	1	2	2	1	2	1	2	2	2	2	2	2	2	3	2	2	1	2	3	4	3	3	2	3	3	2	2	2	2	3	3	3	2
V4	1	1	1	1	0	1	1	1	1	2	2	2	1	1	2	1	2	1	1	1	1	1	2	1	2	2	1	1	2	1	3	3	3	3	2	3	2	2	2	2	2	3	3	2	2
V4t	1	1	1	2	1	0	2	2	1	1	1	1	2	2	2	2	2	1	2	2	2	2	2	2	2	3	2	2	1	2	3	4	3	3	3	4	3	3	2	2	2	3	3	3	2
VOT	2	1	2	2	1	2	0	1	2	2	2	2	2	2	2	2	3	2	1	1	2	2	2	2	3	3	2	2	2	2	3	4	3	3	3	4	3	3	3	3	3	4	4	3	3
VP	2	1	2	1	1	2	1	0	1	1	2	1	1	1	1	1	2	1	2	2	2	2	2	2	2	2	1	2	1	2	2	3	2	2	2	3	2	2	2	2	2	3	3	2	2
MT	1	1	1	1	1	1	2	1	0	1	1	1	1	1	1	2	2	1	2	2	2	2	2	2	2	2	2	2	1	1	2	3	2	2	2	3	2	2	2	2	2	3	3	2	2
MST d/p	2	1	1	1	2	1	2	1	1	0	2	1	1	2	1	1	1	1	2	2	2	2	2	2	1	2	1	2	1	1	2	3	2	2	2	3	2	2	2	2	1	3	3	2	2
MSTl	2	1	2	1	2	1	2	2	1	2	0	1	1	2	1	1	2	1	2	2	2	2	2	3	1	2	2	2	1	2	2	3	2	2	2	3	2	2	2	2	2	3	3	3	2
PO	1	1	1	1	2	1	2	1	1	1	1	0	1	1	1	1	1	2	2	2	3	3	2	3	2	3	2	2	1	2	2	3	2	2	2	3	2	2	2	2	2	3	3	3	2
LIP	2	2	1	1	1	2	2	1	1	1	1	1	0	2	1	1	1	1	2	1	2	2	2	2	2	2	1	2	1	1	2	3	2	2	1	2	2	1	2	1	1	2	3	2	2
PIP	1	1	1	2	1	2	2	1	1	2	2	1	2	0	2	1	1	2	2	2	2	2	2	2	3	3	2	2	2	2	3	3	3	2	3	3	3	2	3	2	3	3	4	3	3
VIP	2	1	1	2	2	2	2	1	1	1	1	1	1	2	0	2	1	1	2	2	3	3	2	3	2	2	2	2	1	1	1	2	1	1	1	2	1	1	1	1	1	2	2	2	2
DP	2	2	2	1	1	2	2	1	2	1	1	1	1	1	2	0	1	1	2	2	2	2	2	2	2	2	2	2	1	1	3	3	3	2	2	3	2	2	2	2	2	3	3	2	2
7a	2	2	2	2	2	2	3	2	2	1	2	1	1	1	1	1	0	1	2	2	2	2	1	2	2	2	1	1	1	1	2	2	2	1	2	2	2	1	2	1	2	2	3	2	2
FST	2	1	1	1	1	1	2	1	1	1	1	2	1	2	1	1	1	0	1	1	2	2	2	2	1	2	1	1	1	2	2	3	2	2	2	3	2	2	2	2	2	3	3	2	2
PITd	2	2	2	2	1	2	1	2	2	2	2	2	2	2	2	2	2	1	0	2	2	1	1	1	2	2	2	2	1	1	3	3	3	3	3	3	2	2	2	2	3	3	3	2	2
PITv	2	2	2	2	1	2	1	2	2	2	2	2	1	2	2	2	2	1	2	0	1	1	1	1	2	2	1	1	1	1	3	3	3	3	2	3	2	2	2	2	2	3	3	2	2
CITd	2	2	2	2	1	2	2	2	2	2	2	3	2	2	3	2	2	2	2	1	0	2	1	1	1	2	2	1	2	2	4	4	4	3	3	4	3	3	3	3	3	3	3	3	2
CITv	2	2	2	2	1	2	2	2	2	2	2	3	2	2	3	2	2	2	1	1	2	0	1	1	1	2	1	1	2	2	4	4	3	3	2	3	3	3	3	3	3	3	3	2	2
AITd	3	2	2	2	2	2	2	2	2	2	2	2	2	2	2	2	1	2	1	1	1	1	0	2	2	1	1	1	1	1	3	3	3	2	2	3	2	2	2	2	3	3	3	2	2
AITv	2	2	2	2	1	2	2	2	2	2	3	3	2	2	3	2	2	2	1	1	1	1	2	0	2	2	1	1	2	2	4	4	3	3	2	3	3	3	3	3	3	3	3	2	2
STPp	3	2	2	2	2	2	3	2	2	1	1	2	2	3	2	2	2	1	2	2	1	1	2	2	0	1	1	1	1	1	3	3	3	3	2	3	2	2	2	2	2	3	3	2	2
STPa	3	3	2	3	2	3	3	2	2	2	2	3	2	3	2	2	2	2	2	2	2	2	1	2	1	0	1	1	2	1	3	3	3	3	2	3	2	2	2	3	3	3	3	2	2
TF	2	2	1	2	1	2	2	1	2	1	2	2	1	2	2	2	1	1	2	1	2	1	1	1	1	1	0	2	2	1	3	3	2	2	1	2	2	2	2	2	2	2	2	1	1
TH	2	2	2	2	1	2	2	2	2	2	2	2	2	2	2	2	1	1	2	1	1	1	1	1	1	1	2	0	2	1	3	3	3	2	3	3	2	2	2	2	3	2	2	2	1
FEF	2	1	1	1	2	1	2	1	1	1	1	1	1	2	1	1	1	1	1	1	2	2	1	2	1	2	2	2	0	1	2	3	2	2	2	3	2	2	1	1	2	2	2	2	1
46	2	2	2	2	1	2	2	2	1	1	2	2	1	2	1	1	1	2	1	1	2	2	1	2	1	1	1	1	1	0	2	2	2	2	2	2	1	1	1	2	2	2	2	1	1
3a	3	2	2	3	3	3	3	2	2	2	2	2	2	3	1	3	2	2	3	3	4	4	3	4	3	3	3	3	2	2	0	1	1	1	2	2	1	2	1	2	2	2	2	3	3
3b	4	3	3	4	3	4	4	3	3	3	3	3	3	3	2	3	2	3	3	3	4	4	3	4	3	3	3	3	3	2	1	0	1	1	2	2	1	2	2	2	2	2	2	3	3
1	3	2	2	3	3	3	3	2	2	2	2	2	2	3	1	3	2	2	3	3	4	3	3	3	3	3	2	3	2	2	1	1	0	1	1	2	1	1	2	1	2	2	2	3	3
2	3	2	2	3	3	3	3	2	2	2	2	2	2	2	1	2	1	2	3	3	3	3	2	3	3	3	2	2	2	2	1	1	1	0	1	2	1	1	1	1	1	2	2	3	3
5	3	2	2	2	2	3	3	2	2	2	2	2	1	3	1	2	2	2	3	2	3	2	2	2	2	2	1	3	2	2	2	2	1	1	0	1	1	1	1	1	1	2	2	2	2
Ri	4	3	3	3	3	4	4	3	3	3	3	3	2	3	2	3	2	3	3	3	4	3	3	3	3	3	2	3	3	2	2	2	2	2	1	0	1	1	2	2	2	1	2	2	2
SII	3	2	2	3	2	3	3	2	2	2	2	2	2	3	1	2	2	2	2	2	3	3	2	3	2	2	2	2	2	1	1	1	1	1	1	1	0	1	1	1	2	1	1	2	2
7b	3	2	2	2	2	3	3	2	2	2	2	2	1	2	1	2	1	2	2	2	3	3	2	3	2	2	2	2	2	1	2	2	1	1	1	1	1	0	1	1	1	1	2	2	2
4	3	2	2	2	2	2	3	2	2	2	2	2	2	3	1	2	2	2	2	2	3	3	2	3	2	2	2	2	1	1	1	2	2	1	1	2	1	1	0	1	1	2	2	2	2
6	3	2	2	2	2	2	3	2	2	2	2	2	1	2	1	2	1	2	2	2	3	3	2	3	2	3	2	2	1	2	2	2	1	1	1	2	1	1	1	0	1	2	2	3	2
SMA	3	2	2	2	2	2	3	2	2	1	2	2	1	3	1	2	2	2	3	2	3	3	3	3	2	3	2	3	2	2	2	2	2	1	1	2	2	1	1	1	0	2	3	3	3
Ig	4	3	3	3	3	3	4	3	3	3	3	3	2	3	2	3	2	3	3	3	3	3	3	3	3	3	2	2	2	2	2	2	2	2	2	1	1	1	2	2	2	0	1	1	1
Id	4	3	3	3	3	3	4	3	3	3	3	3	3	4	2	3	3	3	3	3	3	3	3	3	3	3	2	2	2	2	2	2	2	2	2	2	1	2	2	2	3	1	0	1	1
35	3	3	2	3	2	3	3	2	2	2	3	3	2	3	2	2	2	2	2	2	3	2	2	2	2	2	1	2	2	1	3	3	3	3	2	2	2	2	2	3	3	1	1	0	2
36	3	2	2	2	2	2	3	2	2	2	2	2	2	3	2	2	2	2	2	2	2	2	2	2	2	2	1	1	1	1	3	3	3	3	2	2	2	2	2	2	3	1	1	2	0

Figure 14: The shortest path distance matrix, $\boldsymbol{D}_s$, for the Macaque cortical network [4].

References

[1] Wayne W Zachary. An information flow model for conflict and fission in small groups. *Journal of Anthropological Research*, pages 452–473, 1977. Data Accessed: 2016-07-1 <http://vlado.fmf.uni-lj.si/pub/networks/data./WaFa/default.htm>.

[2] Patrick Doreian, Vladimir Batagelj, and Anuška Ferligoj. Generalized blockmodeling of two-mode network data. *Social Networks*, 26(1):29–53, 2004.

[3] K.-I. Goh, Michael E Cusick, David Valle, Barton Childs, Marc Vidal, and A.-L. Barabasi. The human disease network. *Proceedings of the National Academy of Sciences*, 104(21):8685–8690, may 2007.

[4] László Négyessy, Tamás Nepusz, László Kocsis, and Fülöp Bazsó. Prediction of the main cortical areas and connections involved in the tactile function of the visual cortex by network analysis. *European Journal of Neuroscience*, 23(7):1919–1930, 2006. Data Accessed: 2016-07-1 <https://github.com/igraph/igraphdata>.
